# Prediction of improved antimalarial chemotherapy of artesunate-mefloquine in combination with mefloquine sensitive and resistant *Plasmodium falciparum* malaria

**DOI:** 10.1371/journal.pone.0282099

**Published:** 2023-02-23

**Authors:** Teerachat Saeheng, Kesara Na-Bangchang

**Affiliations:** 1 Center of Excellence in Pharmacology and Molecular Biology of Malaria and Cholangiocarcinoma, Chulabhorn International College, Thammasat University (Rangsit Campus), Klongneung, Klongluang District, Pathumthani, Thailand; 2 Drug Discovery and Development Center, Office of Advanced Science and Technology, Thammasat University (Rangsit Campus), Klongneung, Klongluang District, Pathumthani, Thailand; Menzies School of Health Research, AUSTRALIA

## Abstract

**Background:**

Declining in susceptibility of *Plasmodium falciparum* to mefloquine is reported in South-East Asia. A revisiting on mefloquine pharmacokinetics-pharmacodynamics (PK/PD) could assist in finding new appropriate dosage regimens in combination with artesunate as a three-day course treatment.

**Objective:**

This study aimed to investigate promising alternative artesunate-mefloquine combination regimens that are effective for the treatment of patients with mefloquine-sensitive and resistant *P*. *falciparum* malaria.

**Methods:**

Data collected during 2008–2009 from 124 patients with uncomplicated *P*. *falciparum* malaria were included in the analysis, 90 and 34 patients with sensitive and recrudescence response, respectively. All patients were treated with a three-day combination of artesunate-mefloquine. Population PK-PD models were developed. The developed models were validated with clinically observed data. Simulations of clinical efficacy of alternative mefloquine regimens were performed based on mefloquine sensitivity, patients’ adherence and parasite biomass.

**Results:**

The developed PK/PD models well described with clinically observed data. For mefloquine-resistant *P*. *falciparum*, a three-day standard regimen of artesunate-mefloquine is suitable (>50% efficacy) only when the level of parasite sensitivity was < 1.5-fold of the cut-off level (IC_50_ < 36 nM). For mefloquine-sensitive parasite with IC_50_ < 23.19 nM (0.96-fold), all regimens provided satisfactory efficacy. In the isolates with IC_50_ of 24 nM, regimen-I is recommended. Curative treatment criteria for mefloquine and artesunate were C_336h_ (>408 ng.mL^-1^) or C_max_/IC_50_ (>130.1 g.m/M), and C_max_/IC_50_ (>381.2 g.m/M), respectively.

**Conclusions:**

Clinical use of a three-day standard artesunate-mefloquine is suitable only when the IC_50_ of *P*. *falciparum* isolates is lower than 36 nM. Otherwise, other ACT regimens should be replaced. For mefloquine-sensitive parasite, a dose reduction is recommended with the IC_50_ is lower than 23.19 nM.

## Introduction

Malaria-related mortality has tremendously reduced during the last two decades since the introduction of artemisinin-based combination therapy (ACT) [1]. Artesunate-mefloquine is one of the commonly used ACT for the first-line treatment of uncomplicated *Plasmodium falciparum* in Southeast Asia and Africa [2–18]. In South-East Asia, its clinical efficacy has been continuously declined, with failure rates of 10–30% [1]. The highest failure rate was reported in the Thai-Myanmar border areas (42-day cure rate of 72.58%) in 2010 [18]. This high failure rate was attributed to pharmacokinetic factors (inadequate drug concentrations) and reduced susceptibility or resistance of *P*. *falciparum* to either mefloquine or artesunate, or both. Inadequate blood concentrations of mefloquine was shown to significantly influence treatment response compared with artesunate. Notably, the current three-day course regimen may not provide adequate drug concentrations in the resistant parasites. Revisiting the pharmacokinetic and pharmacodynamic relationship of drug concentration-time profile and clinico-parasitological response following this combination therapy may offer effective alternative dosage regimens for both mefloquine-sensitive and resistant *P*. *falciparum*. The pharmacokinetic-pharmacodynamics (PK/PD) modelling has been successfully applied to predict the appropriate dosage regimens of various antimalarial drugs for a clinical use [19–23], exemplified by the SJ733 (an oral ATP4 inhibitor) [23]. The current study aimed to investigate promising alternative regimens of artesunate-mefloquine with improved efficacy to cope with multidrug resistant *P*. *falciparum* using PK/PD model approach.

## Materials and methods

**Fig 1:**
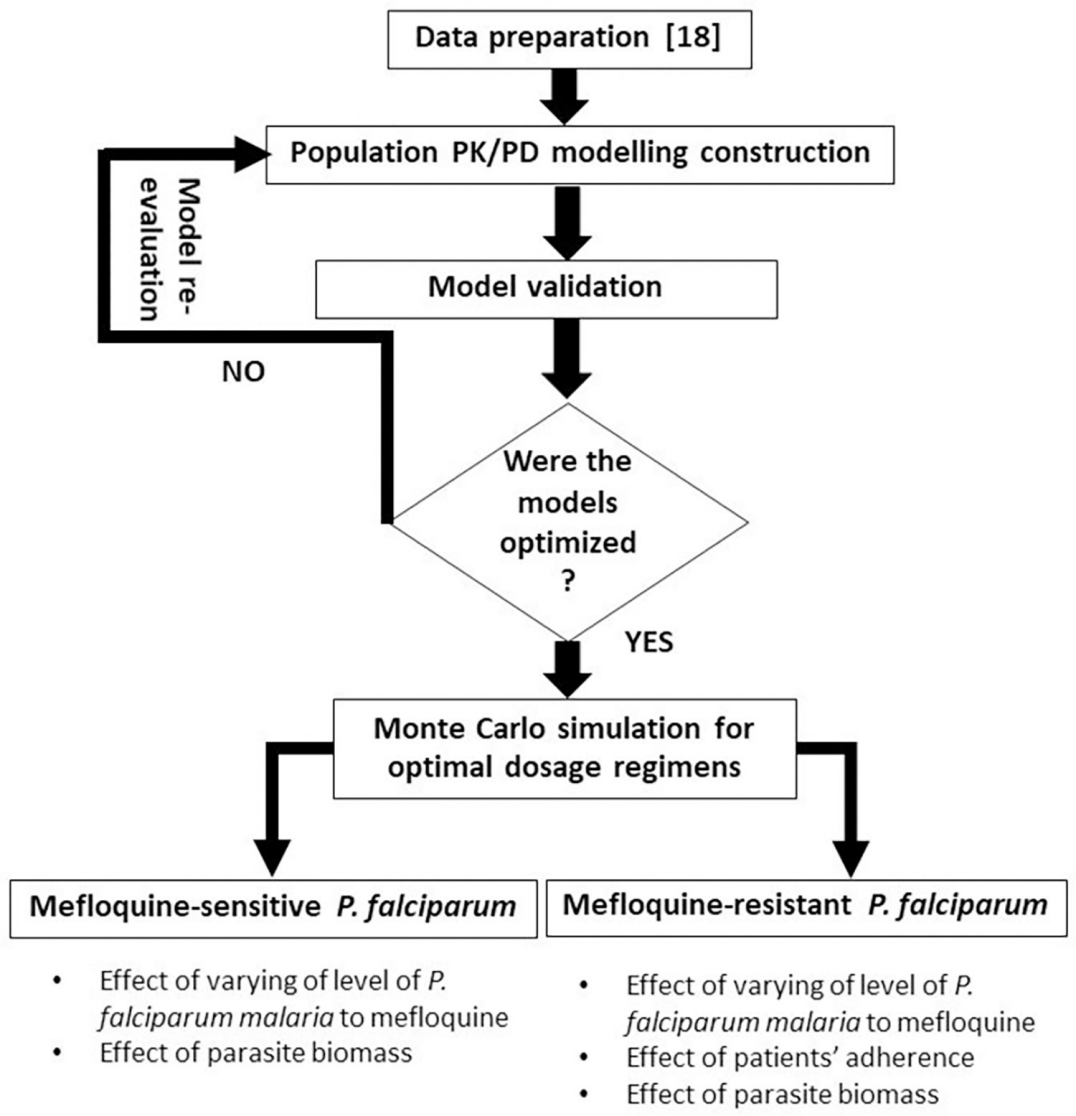
A flow-chart diagram of materials and methods.

The flow-chart of study framework is shown in **Fig 1.**

### Data source and study population

Data from the previously published article on the clinical efficacy of the three-day artesunate-mefloquine combination in the Thai-Myanmar borders during 2008–2009 were used for pharmacokinetic-pharmacodynamic (PK/PD) analysis [18]. All patients were diagnosed with uncomplicated *P*. *falciparum* malaria. In brief, 124 patients (aged 16–50 years) were included in the study, 90 and 34 patients with sensitive and recrudescence response, respectively. All received 200 mg of artesunate and 750 mg of mefloquine on day 1, followed by 200 mg of artesunate, and 500 mg of mefloquine on day 2, followed by 30 mg of primaquine on day 3.

### Population PK/PD modeling

The PK/PD models for mefloquine and artesunate/dihydroartemisinin (active metabolite of artesunate) were constructed using nonlinear mixed-effects modeling (MonolixSuite software, version 2021R1, Antony, France; Lixoft SAS, 2021). Pharmacokinetic parameters were estimated using the build-in stochastic approximation expectation-maximization algorithm. Various compartment model with different order absorption and elimination were performed to fit with drug concentration-time data. Pharmacokinetic parameters were normally distributed when transformed to log-scale. The pharmacodynamic model was evaluated using E_max_ model (turn-over rate) with production inhibition. The model was corrected with the fraction of unbound drug (f_u_) in plasma and tissue and was tested for sigmoidicity characteristics. Pharmacodynamic parameters were tested following non-transformed and log-normal transformation. The pharmacodynamic equation is shown below;

$$\frac{d(parasite)}{d(t)}=K_{in}*\left(\frac{1-C*fu}{C*fu+{IC}_{50}*fu}\right)-K_{out}*parasite$$ (1)

Where parasite is number of parasite; C is drug plasma-concentration profiles; IC_50_ is the half maximal inhibitory concentration that inhibit parasite growth by 50%; K_in_ is indirect turnover model with full inhibition of production; K_out_ is the degradation rate of parasite. The residual variability and the types of error models were evaluated using proportional, constant, and combined error models with power law. Predefined criteria for a model selection were: (i) the decrease in minimum objective function value (OFV), Akaike Information Criteria (AIC), Bayesian Information Criteria (BIC), and Corrected Bayesian Information Criteria (BICc), and (ii) the percentage of root mean square errors (RSE%), Graphical Goodness of Fit (GOF) including the observed *versus* predicted concentrations, scatter plot of residual, and the virtual predictive check (VPC). A significant level for the inclusion of the covariates (age, sex, bodyweight, and mefloquine level before treatment) in the model was set at α = 0.05.

The plotting of model-based individual prediction (IPRED) and population prediction (PRED) *versus* observed concentrations (GOF) was used for model evaluation. VPC included the observational data *versus* simulated data (1,000 patients) with the 10th, 50th, and 90th percentiles.

### PK/PD model validation

The IC_50_ (concentration that inhibits parasite growth by 50%) reflecting parasite’s susceptibility to mefloquine from the clinical study in the same areas areas [24] were used for model validation (IC_50_ 12.9, 19.1 and 30.4 nM for isolates from Ranong, Kanchanaburi and Ratchaburi, and Tak provinces, respectively). These IC_50_ values were used to report the drug’s efficacy in those area (Ranong, Kanchanaburi and Ratchaburi, and Tak province). The evaluation of drug’s efficacy were described in the next section. The pharmacodynamic endpoint were the 42 days curative rate.

### Monte Carlo simulations

The final PK/PD (1,000 virtual patients with 10 simulations) models were used to simulate optimal dosages of mefloquine in the combination regimens that provided high clinical efficacy using Monte Carlo simulations (Simulix version 2021R1, Antony, France; Lixoft SAS, 2021). The simulated dose regimens (oral administration) for mefloquine-resistant *P*. *falciparum* included: (i) 750 mg on day 1 (day 0), followed by 500 mg on day 2, (ii) 500 mg once daily for 42 days, (iii) 500 mg every 72 hours for 42 days, (iv) 500 mg every 96 hours for 42 days, and (v) 250 mg every 12 hours for 42 days. The simulated regimens for mefloquine-sensitive strains were: (i) 750 mg on day 1 and 500 mg on day 2, (ii) 500 mg on day 1 and 250 mg on day 2, (iii) 750 mg on day 1, (iv) 500 mg on day 1, and (v) 250 mg on day 1, 2, and 3. The simulated regimens are based on trials and erros until it provides the curative rate close to 100%.

### Effect of *P*. *falciparum* susceptibility to mefloquine

The cut-off level for mefloquine sensitivity used was 24 nM [20,21]. Simulations were performed using five simulated levels of mefloquine-resistant *P*. *falciparum* based on the current IC_50_ (23.19 nM) of sensitive strains, *i*.*e*., IC_50_: 24 nM (1-fold of the cut-off level), 30.4 (1.25-fold), 36 (1.5-fold), 48 (2-fold), 72 (3-fold), and 120 (5-fold) nM. The IC_50_ used for the sensitive strains were: 6 (0.25-fold), 12 (0.5-fold), 12.9 (0.54-fold), 19.1 (0.8-fold), and 23.19 (0.96-fold) nM. In addition, the IC_50_ reported in 2010 in the current study [18] of 23.19 (mefloquine-sensitive) and 63.84 (mefloquine-reistant) nM were also used in the simuluation. Mefloquine-sensitive and recrudescence *P*. *falciparum* responses refer to adequate clinical and parasitological response at day 42 (ACPR42) as a primary endpoint based on the previous study [18].

### Effect of patients’ adherence

Due to long course treatment of the proposed reimen (42 days), the effect of patients’ adherence to medication (*i*.*e*., 100%, 70%, 50%, and 30%) on treatment efficacy were evaluated.

### Effect of admission parasite biomass

The effect of parasite biomass (30,000, 20,000, 15,000 and 5000/μL) on the efficacy of mefloquine in the standard regimen (regimen-I) was evaluated using the cut-off level for curative treatment of < 10/μL on day 42) [25]. Treatment outcome was classified as low (<50% cure), moderate (50–70% cure), and high (>70% cure) efficacy [23]. Statistical analysis (relative risk, odds ratio, Fisher’s exact and chi-square tests) were performed at a significance level (α) of 0.05.

### Toxicity approach

Due to the permanent neurological deficits following standard treatment, the relative risk of plasma/blood concentration over the threshond, resulting in the disruption of calcium homeostasis and ER function following five regimens, were calculated.

### Establishment of the criteria for curative treatment

Receiving Operating Characteristic (ROC) curves were used to assess the accuracy of the following cut-off parameters for curative outcome: area under the curve during the first 7 days (AUC_0-7days_), trough plasma/whole-blood concentration at 336 h (C_trough,336h_), and peak concentration and IC_50_ ratio (C_max_ /IC_50_). It is noted that the mechanism of action of a mefloquine is on the blood stage (blood schizontocide), curative parameter “C_trough, 336h_ (14 days)” covers the blood stage. A binomial proportion (Wilson/Brown) method was performed at a statistically significant level of 0.05 (GraphPad Prism version 9.20 for Windows, GraphPad Software, La Jolla California USA). Sensitivity, specificity, accuracy, negative predictive value (NPV), positive predictive value (PPV), positive likelihood ratios (LR+), negative likelihood ratios (LR-), and diagnostic odd ratios were calculated for an internal validity.

## Results

### PK/PD models

A one-compartmental model with zero-order kinetic absorption and linear elimination showed the best characterized (best fit) the population pharmacokinetic properties for mefloquine. A transit-compartment model with one compartment and linear elimination best characterized (best fit) the pharmacokinetic properties of artesunate and dihydroartemisinin. The pharmacokinetic model of artesunate/dihydroartemisinin was in accordance with that previously reported [a transit-compartment model followed by one-compartment disposition] [26]. Since both artesunate and dihydroartemisinin are almost immediately eliminated from the systemic circulation, their contribution to parasite elimination is only during the first two days of treatment. Therefore, the neither artesunate nor artesunate could be fit with parasite clearance throughout 42 days. With a short-half-life, primaquine unlikely plays important role for parasite elimination due to fast elimination, particulary of blood stage. Only mefloquine was therefore used for PK/PD modeling and simulation as it play role in parasite elimination for the whole 42-day follow up. The final PK/PD model of mefloquine was well characterized by E_max_ model (turn-over rate) without sigmoidicity when corrected with f_u_. The inclusion of body weight and sex did not improve model quality. All parameters in all models showed low to moderate variation (%RSE). The analysis of OFV, AIC, BIC, BICc and GOF were summarized in the **S1 File**. Final population parameters estimated for mefloquine (resistance and sensitive), artesunate and dihydroartemisinin are shown in **Tables 1 and 2,** respectively.

**Table 1:** Pharmacokinetics/pharmacodynamics parameters.

Sensitive-mefloquine				Resistant-mefloquine		
**Fixed effects**						
**Parameters**	**Mean**	**SE**	**%RSE**	**Mean**	**SE**	**%RSE**
**Tk_pop**	6.05	0.47	7.78	9.83	1.85	18.8
**V_pop**	155915.5	6232.5	4.00	162500	20800	12.9
**K_pop**	0.0028	0.00025	9.02	0.0029	0.0005	17.8
**R**_**0**_ **_pop**	7649998.41	11.13	0.000146	10600000	2060000	19.4
**K**_**out**_ **_pop**	0.13	0.0044	3.48	0.092	0.0061	7.33
**IC**_**50**_ **_pop**	3.41	2.12	62.1	29.5	11.6	39.1
**Error model parameters**						
**A**	46.21	13.28	28.7	161	73.1	45.4
**B**	0.13	0.011	8.40	0.162	0.046	28.3
**aParasite**	102394.71	19051.99	18.6	192000	69100	35.9
**bParasite**	0.39	0.03	7.62	0.295	0.0629	21.3

**Table 2:** Pharmacokinetics parameters.

Artesunate				Dihydroartemisinin (DHA)		
**Fixed effects**						
**Parameters**	**Mean**	**SE**	**%RSE**	**Mean**	**SE**	**%RSE**
**K**_**tr**_ **_pop**	14.9	1.44	9.69	1.18	0.19	16.3
**Mtt_pop**	1.36	0.085	6.21	3.17	0.069	2.19
**K**_**a**_ **_pop**	31.74	4.96	15.6	12.75	1.08	8.47
**Cl_pop**	50909.09	17472.02	34.3	10238.33	2520.41	24.60
**V**_**1**_ **_pop**	84.89	40.45	47.6	1.82	0.69	37.7
**Q**_**2**_ **_pop**	12.33	1.69	14.9	59.56	29.88	50.2
**V**_**2**_ **_pop**	0.096	0.015	15.9	0.083	0.013	15.9
**Q**_**3_**_ **pop**	9.54	1.06	11.1	23	4.57	19.90
**V**_**3**_ **_pop**	0.24	0.03	12.5	0.75	0.22	29.2
**Error model parameters**						
**b**	0.89	0.061	6.84	0.75	0.044	5.79

### Model validation

The developed PK/PD models using the IC_50_ of 12.9, 19.1 and 30.4 nM adequately predicted the reported clinical efficacy of mefloquine [19]. The efficacy predicted based on the IC_50_ of 23.19 (sensitive) and 63.84 nM (resistance) reported for the current study was 74 and 13.8%, respectively [18].

### PK/PD simulations for prediction of the clinical efficacy of mefloquine

#### Mefloquine-resistant *P*. *falciparum*

**Fig 2:**
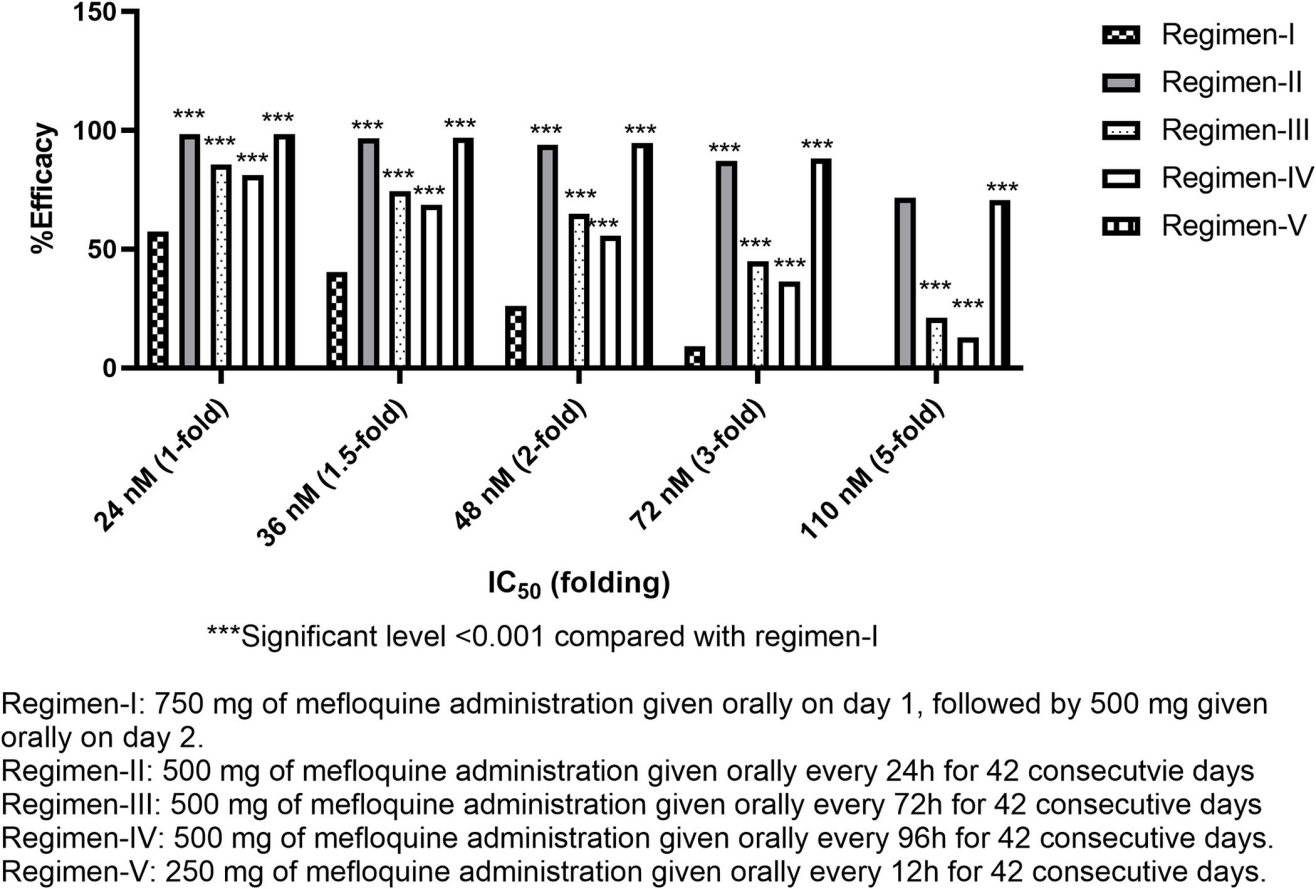
Comparisons of treatment efficacy of different proposed regimens and IC50 values with 100% of adherence in patients with resistant mefloquine.

**Fig 3:**
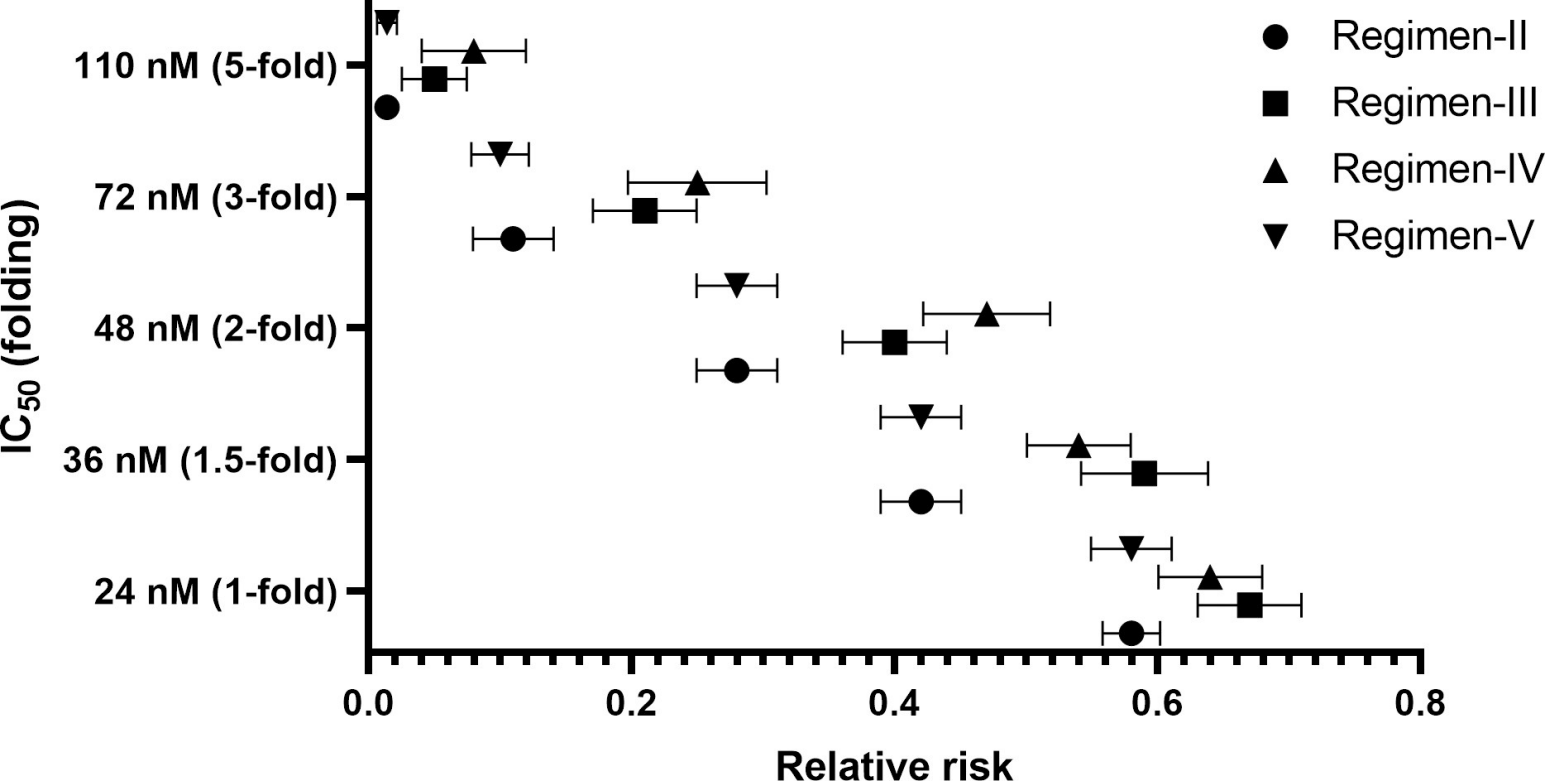
Comparisons of relative riskof different proposed regimens and IC50 values with 100% of adherence in patients with resistant mefloquine.

**Fig 4:**
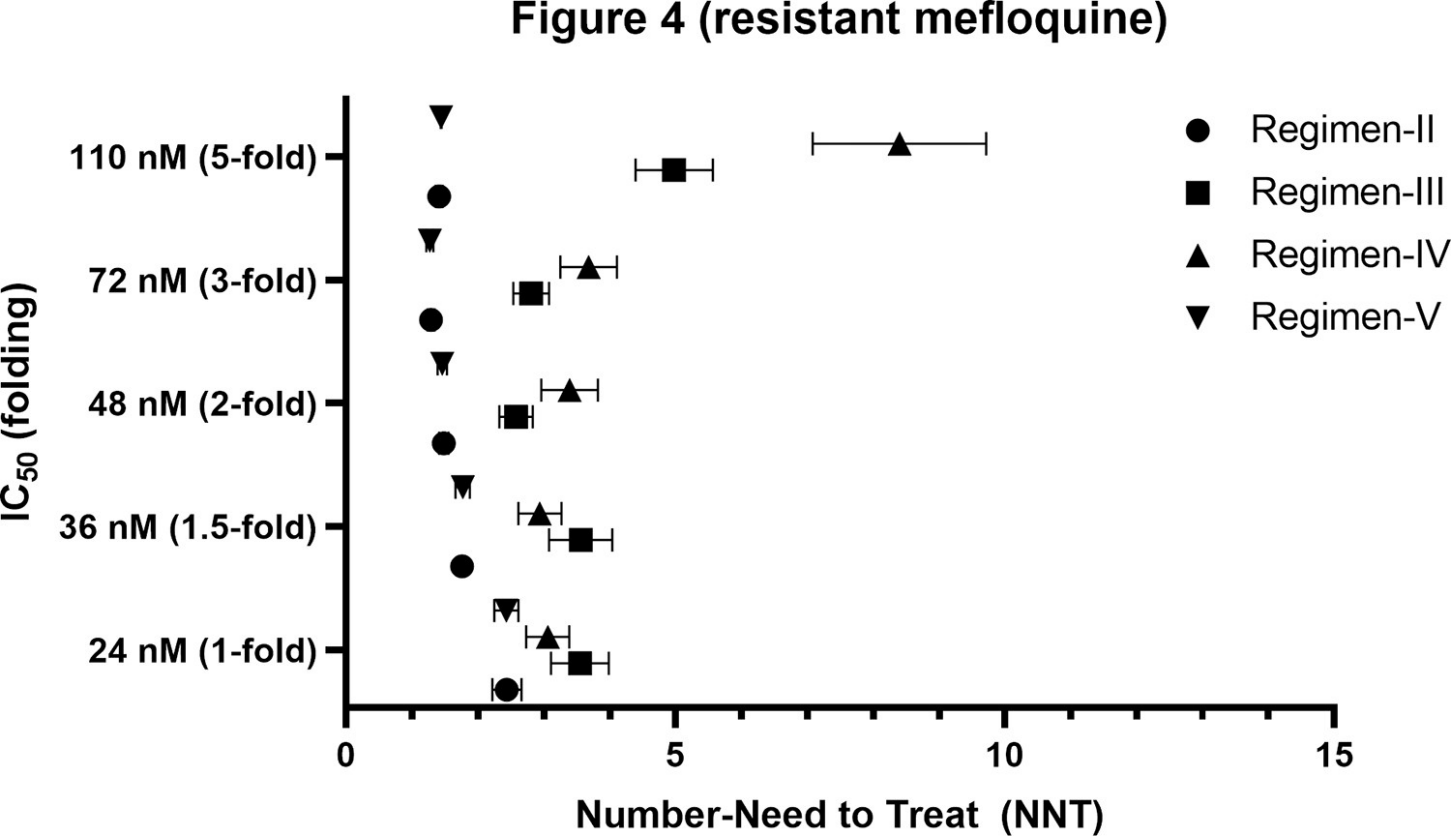
Comparisons ofnumber-need to treat of different proposed regimens and IC50 values with 100% of adherence in patients with resistant mefloquine.

Without the effect of patients’ adherence (100%), the efficacy of mefloquine for regimen-I (standard regimen) ranged from 1 to 57.3% (**Fig 2**), with IC_50_ ranging from 1 to 5-fold (24–110 nM). All other regimens provided better treatment efficacy than regimen-I (p ≤ 0.001). Regimen-V was the most effective (70.6–98.4% cure) (**Fig 2**). Number-needed-to-treat (NNT) and relative risk (RR) for regimen-V were 1.44–2.43 and 0.04–0.58, respectively. With a 1 to 2-fold increase in IC_50_ (24–72 nM), all proposed regimens provided moderate efficacy (>50%). With a 3 to 5-fold increase in IC_50_, the efficacy of regimen-III and IV dramatically dropped to around 30 and 15%, respectively. The efficacy of regimen-II and V were high (>70%). The simulated C_max_ ratios between regimen-II, III, IV and V compared with regimen-I were 5.65, 1.82, 1.44, and 5.55-fold, respectively. Since regimen-II and V provided C_max_ over 5-fold of the standard regimen, patients may be at risk of toxicities. Regimen-II and V were inappropriate for clinical use, and therefore, the effect of patients’ adherence was evaluated only for regimen-III, and IV. Treatment efficacy, RR, and NNT for all regimens when the adherence was 100% are presented in Figs 2–4 (2, 3, and 4 for efficacy, RR, and NNT, respectively). With 70% adherence, regimen-III and IV provided improved efficacy compared with regimen-I for isolates with different levels of sensitivity to mefloquine (p<0.001) for mefloquine resistance (**S1 File**).The NNT and RR were 3.21–43.68 and 0.12–0.81, respectively. When the IC_50_ was 1 to 1.5-fold of the cut-off value, the efficacy of regimen-III and IV were moderate (50–70%) and high (>70%), respectively. When the IC_50_ was increased by 2-fold, regimen-IV provided low efficacy (43.9%) efficacy. When the IC_50_ was increased by 5-fold, the efficacy for regimen-III and IV were 13.3 and 7.2%, respectively (**S1 File**).

With 50% adherence, both regimen-III and IV still provided superior efficacy than regimen-I (α = 0.001). Only a 1-fold increase of IC_50_ resulted in moderate efficacy (>50%) for both regimen-III and IV. When the IC_50_ was increased by 1.5-fold, treatment efficacy of regimen-III was still over 50% (NNT = 5.05, p<0.001). With a 2 to 3-fold increase in IC_50_, the efficacy was 15–50% for both regimens. With a 5-fold increase in IC_50_, the efficacy of both regimens were lower than 10%.

**Fig 5:**
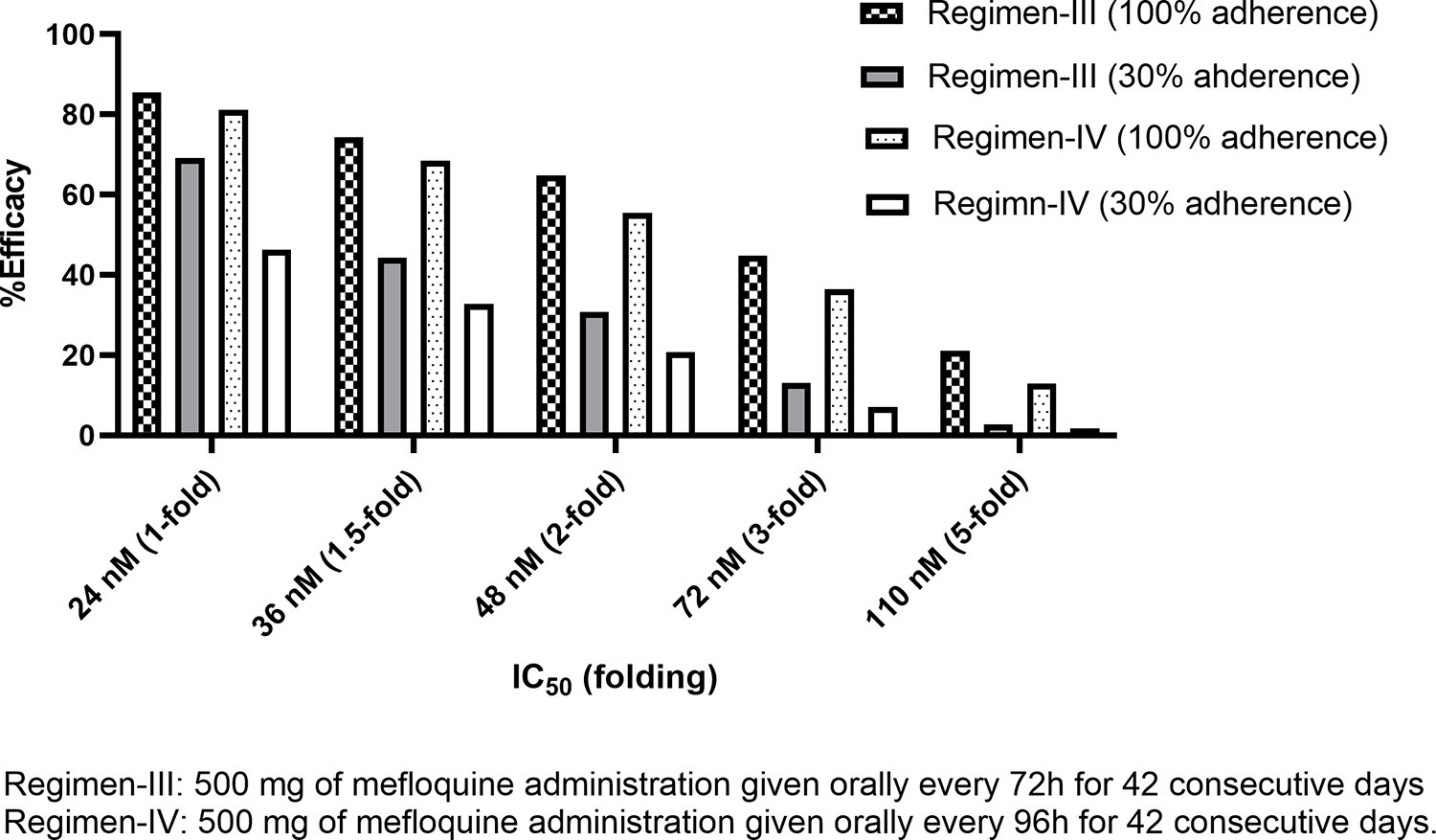
Comparisons of treatment efficacy of two propose regimens (III, and IV) between 100% of adherence and 30% of adherence in patients with resistant mefloquine.

**Fig 6:**
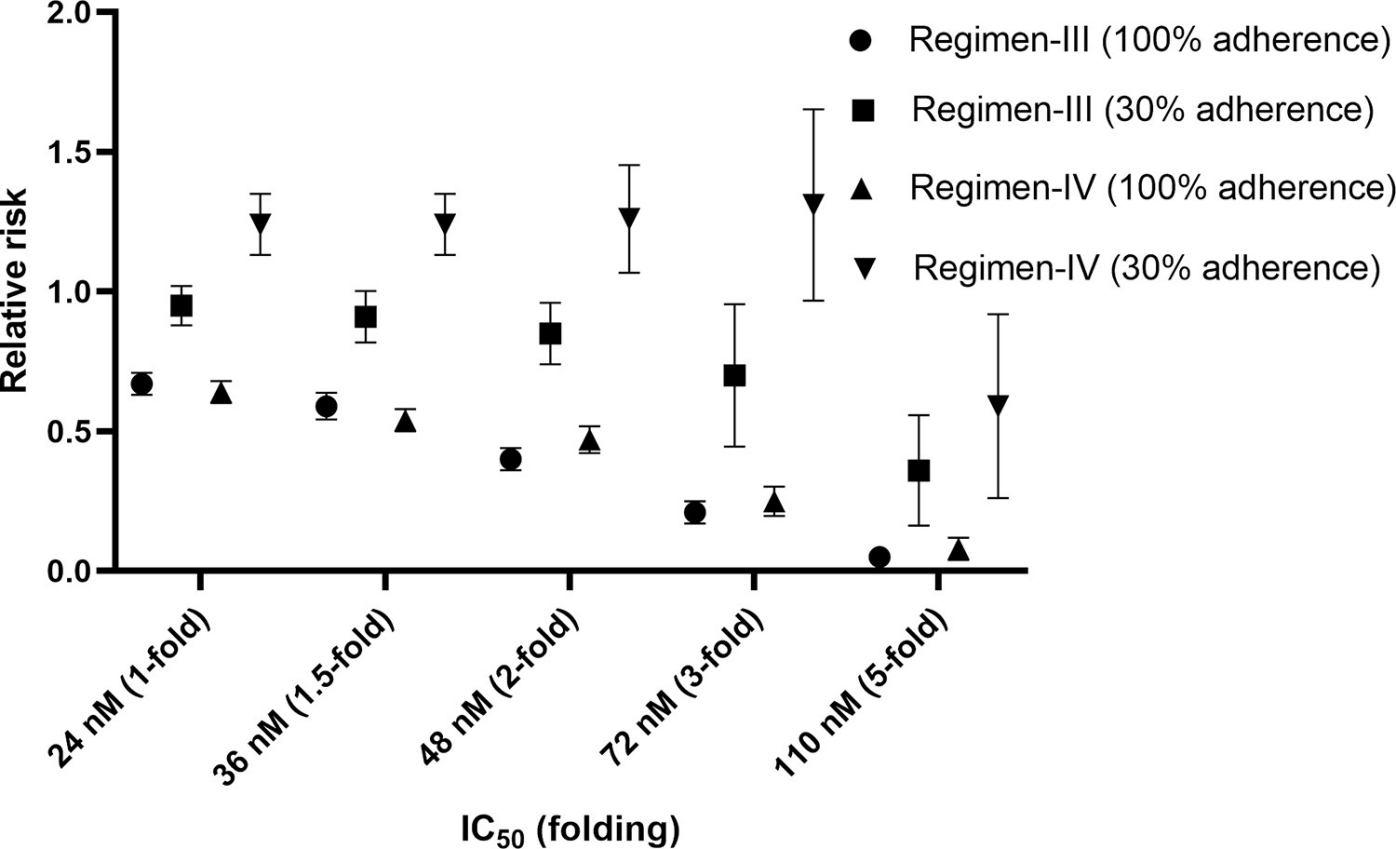
Comparisons ofrelative risk of two propose regimens (III, and IV) between 100% of adherence and 30% of adherence in patients with resistant mefloquine.

With 30% adherence, the efficacy of regimen-III appeared to be slightly higher than regimen-I (**S1 File**). However, no significant difference was found for 1-fold (p = 0.2), 1.5-fold (p = 0.09), and 2-fold (p = 0.02) increase in IC_50_. The efficacy of regimen-IV for all sensitivity levels except 5-fold was lower than regimen-I. No significant difference was found between regimen-I and IV when the IC_50_ was increased by 3-fold (p = 0.08) and 5-fold (p = 0.24). The efficacy and RR of each regimen at 100 and 30% adherence are shown in **Figs 5 and 6**. The predicted mefloquine plasma/blood concentration profiles for each regimen are shown in **S1 File.**

In addition to toxicity approach, the RR of mefloquine concentration over the threshold of inducing the disruption of calcium homeostasis and ER function for regimen-II, III, IV, and V with 100% of adherence were 3.72 (3.30–4.16), 2.67 (2.37–3.00), 2.29 (2.02–2.59), and 3.67 (3.28–4.11), respectively.

**Fig 7:**
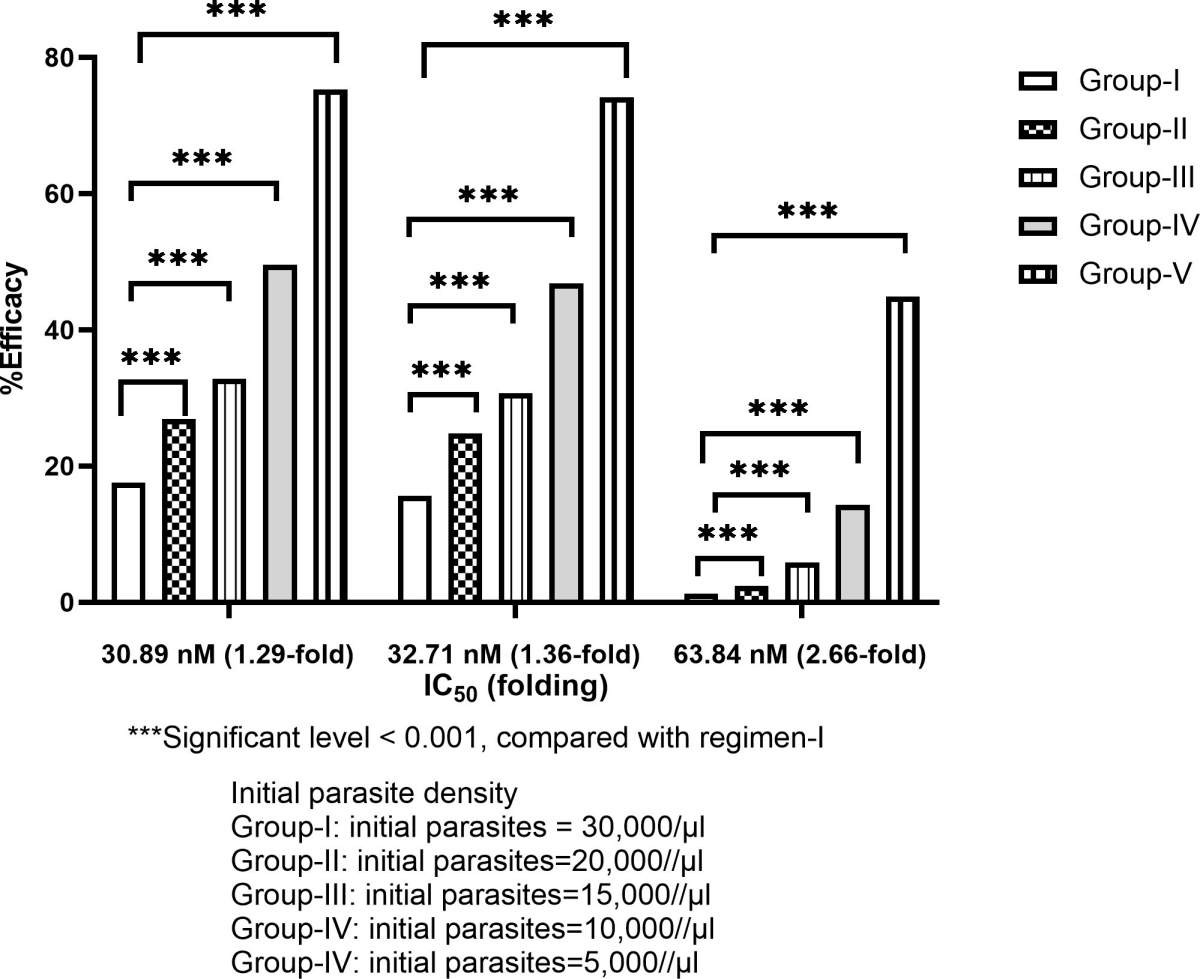
Comparisons of effect of initial parasites density on treatment efficacy of regimen-I (a three-day combination of artesunate-mefloquine) with different IC50 values in patients with resistant mefloquine.

**Fig 8:**
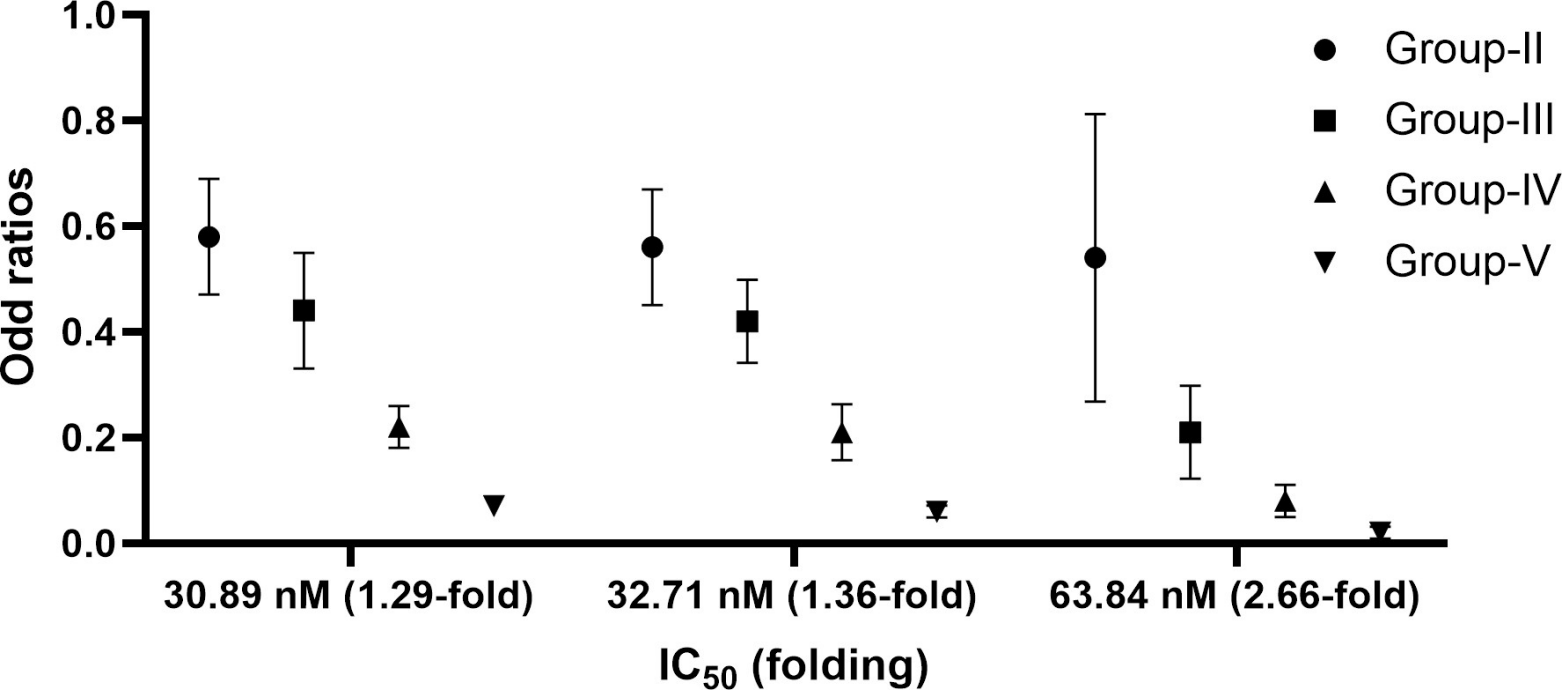
Comparisons ofodd ratios of regimen-I (a three-day combination of artesunate-mefloquine) with different IC50 values in patients with resistant mefloquine.

Five different levels of parasite biomass [*i*.*e*., 30,000 (group-I), 20,000 (group-II), 15,000 (group-III), 10,000 (group-IV), and 5,000 (group-V)] with three different levels of parasite sensitivity to mefloquine were simulated. Overall, treatment efficacy seemed to be an inverse relationship with parasites biomass. All scenarios in all groups except for the IC_50_ of 63.83 nM in group-II (p = 0.1) showed a significant difference in efficacy compared with group-I (p<0.001) (Fig 7), with odds ratios of 0.02–0.58 (**Fig 8**). For all parasite sensitivity levels, the group-V with IC_50_ of 30.89 nM was the most effective regimen (75.30% cure). In contrast, the group-I with IC_50_ of 63.84 nM was the least effective (1.3% cure).

### Mefloquine sensitive *P*. *falciparum*

**Fig 9:**
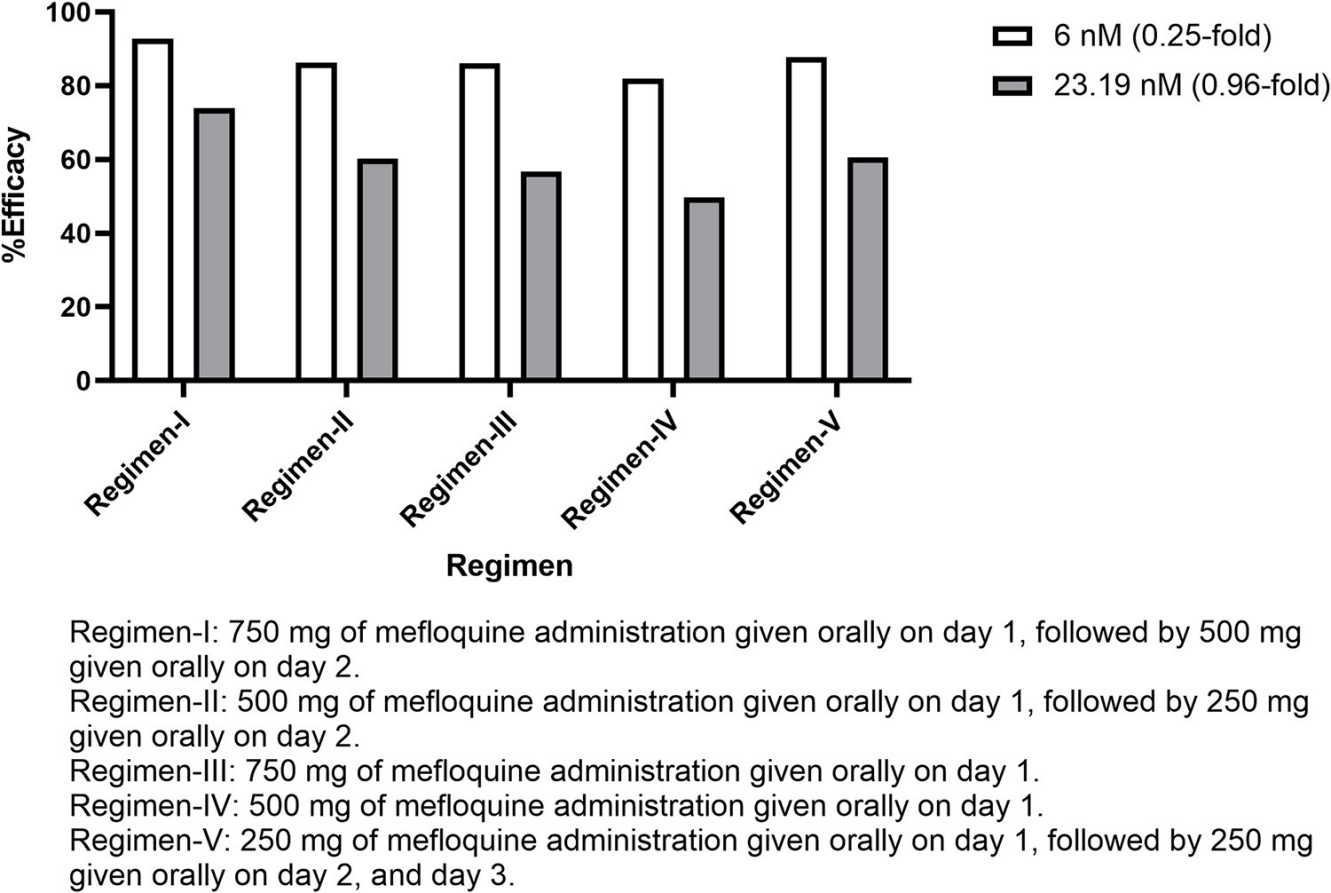
Comparisons of treatment efficacy of five proposed regimens between two IC50 values in patients with sensitive mefloquine.

Three levels of mefloquine sensitivity based on clinical IC_50_ and two varied IC_50_ values were simulated assuming 100% patients’ adherence. Overall, the efficacy of all regimens was inferior to regimen-I for all scenarios (p = 0.001). However, the efficacy of all regimens except regimen-IV with IC_50_ of 23.19 nM (49.7%) were still higher than 50% (moderate efficacy). With the most sensitive strains (IC_50_ 6 nM or 0.25-fold), all regimens provided efficacy over 80% (92.7, 86.3, 86.1, 81.9 and 87.8% for regimen-I, II, III, IV and V, respectively). When the IC_50_ was increased to 12 nM, the efficacy was slightly decreased, but still over 70% (84.5, 75.4, 73.3, 65.8 and 76.5%). With a slight increase in IC_50_ (13.10 nM), the efficacy was still maintained (83.5, 73.4, 71.5, 63.0 and 75.1%. With moderate level of mefloquine sensitivity (IC_50_ 19.39 nM), the efficacy was slightly dropped by 10% (77.1, 65.0, 61.6, 53.7 and 64.6%). Notably, treatment efficacy was dramatically decreased to around 60% when the IC_50_ was increased to 23.19 nM (73.9, 60.2, 56.7, 49.7 and 60.6%). Comparison of clinical efficacy of regimen-I and other regimens for all IC_50_ are provided in **S1 File.** Comparison of treatment efficacy between the 0.25-fold and 0.96-fold IC_50_ with different regimens are shown in **Fig 9**. The predicted mefloquine plasma/blood concentration profiles for each regimen are shown in **S1 File.**

The effect of parasite biomass on the treatment efficacy of the current standard regimen was simulated using the IC_50_ reported in the study [18]. An inverse relationship was observed between parasites biomass and treatment efficacy. With the IC_50_ of 13.10 nM, the efficacy of mefloquine for group-I, II, III, IV and V were 41.9, 54.3, 58.6, 71.9 and 84.9%, respectively. When the IC_50_ was increased to 19.39 nM, the corresponding efficacy was 31.1, 43.2, 49.4, 61.4 and 78.5%, respectively (**S1 File)**

### Curative treatment criteria

ROC curves for mefloquine based on C_trough,336h_ (>408 ng/mL) and C_max_/IC_50_ (>130.1 g/mM) were 0.756 (95%CI: 0.60–0.91, p = 0.006), and 0.75 (95%CI: 0.60–0.91, p = 0.006), respectively. ROC curve for artesunate based on C_max_/IC_50_ (>381.2 g/mM) was 0.75 (95%CI: 0.57–0.93, p = 0.019). Sensitivity, specificity, accuracy, NPV, PPV, LR+, LR-, diagnostic odd ratios are summarized in **Table 3**.

**Table 3:** Model parameters for internal validation.

Parameters	Mefloquine		Artesunate
	C_336h_	C_max/_IC_50_	C_max_/IC_50_
**Sensitivity**	73%	81%	71%
**Specificity**	69%	87%	73%
**Accuracy**	71%	83%	71%
**Negative predictive value (NPV)**	0.61	0.72	0.53
**Positive predictive value (PPV)**	0.79	0.91	0.85
**Positive likelihood ratios (LR+)**	2.34	6.02	2.60
**Negative likelihood ratios (LR-)**	0.39	0.22	0.40
**Diagnostic odd ratios**	5.97	27.3	6.48

## Discussion

The emergence and spread of mefloquine resistance have led to a decrease in clinical efficacy of artesunate-mefloquine combination. For mefloquine-resistant *P*. *falciparum*, a three-day artesunate-mefloquine regimen (regimen-I) should be replaced by other regimens when the IC_50_ of mefloquine was higher than 36 nM (1.5-fold of the cut-off level) (<50% efficacy). It was clear that regimen-II/V provided the best efficacy for *P*. *falciparum* with different sensitivity. These regimens were the best option for mefloquine-resistant *P*. *falciparum*. Nonetheless, these proposed regimens resulted in a 5-fold increase in C_max_ compared with regimen-I. Clinical use of regimen-II or V may result in an increased risk of mefloquine toxicity. In such case, regimen-III or IV would be a preferable choice for mefloquine-resistant parasite with IC_50_ lower than 48 nM, particularly when supervised medication or directly observed therapy (DOT) is applicable. Notably, it is clear that patients’ adherence significantly affected the treatment efficacy of the long-course treatment regimens (42 days). Treatment efficacy for parasite strains with IC_50_ below 48 nM dramatically dropped to lower than 50% when the adherence was only 30%. Interestingly, initial parasite biomass significantly affected the clinical efficacy of mefloquine in the standard regimen (I), the higher initial parasite biomass, the higher treatment failure rate. The current standard regimen is thus, only suitable when the initial parasite biomass was lower than 5,000/μL (IC_50_: 30.89–32.71 nM). For mefloquine-sensitive parasite, although the clinical efficacy of all proposed regimens was relatively low compared with regimen-I (moderate efficacy).

Ideally, regimen-IV with the lowest mefloquine total dose (500 mg) would be a preferable choice considering the amount of dosage administration. This regimen, however, provided the lowest efficacy compared with others. Its efficacy was also decreased to 50% for the parasite strains with IC_50_ of 23.19 nM (**Fig 5**). Regimen-II, III and V had a comparable amount of mefloquine dose as well as clinical efficacy. In cases when patients’ adherence to medication is of great concern, a single dose regimen-III (on day 1) is a preferable choice. Due to Mefloquine-induced neurotoxicity, low dose regimens, *i*.*e*., regimen-II and V are preferable choices. Remarkably, the efficacy of all proposed regimens for the parasite strains with IC_50_ of 23.19 nM was decreased to 60%. In such case, Regimen-I is more appropriate. Similarly to the resistant strains, initial parasite biomass significantly influenced the efficacy of mefloquine in the sensitive isolates. When the IC_50_ of mefloquine was between 13.10 and 19.39 nM, regimen-I was still effective with an initial parasite biomass of lower than 10,000/μL. In cases when the initial parasite density was over 10,000/μL, clinical use of this regimen may not be effective (<50% efficacy).

Besides the problem with mefloquine-resistant *P*. *falciparum* in the Great Mekong Subregion (GMS), the emergence and spread of resistance to artemisinin-based therapy is the great concern in all malaria-endemic areas. The combination of two partner drugs with different mechanisms of action (triple artemisinin-based combination therapy or TACT) is an optional choice for malaria treatment [27,28]. It is noted that this approach has been successfully applied in HIV as well as tuberculosis [27]. The first clinical trial of TACT showed that TACT could tackle artemisinin-based resistance problems with high efficacy, safety and tolerability [28]. TACT is, therefore, a promising approach for malaria therapy.

Few clinical information have been corrected based on previous study [18], therefore, the effects of covariate parameters on models are limited. Since the highest mefloquine resistance level is reported in Thailand [1], the proposed curative criteria based on clinical data in Thailand could be applied for the treatment of uncomplicated *P*. *falciparum* malaria in other endemic areas. The efficacy reported in this study [18] was 78.5%, while the efficacy in other Southeast Asia countries (Cambodia, Myanmar, and Laos) [4–12], and African countries (Mali, and Senegal) [13–17] ranged 96 to 100%. However, clinical application based on the results of the current study may be limited as the PK/PD models did not include the effect of artesunate and dihydroartemisinin on parasite clearance. Furthermore, the impact of initial parasite biomass on the clinical efficacy of regimen-I artesunate-mefloquine combination seemed to be underestimated (low efficacy). An increase in initial parasite biomass has been reported to result in delayed parasite clearance [29]. Therefore, simulation of efficacy for the 42-day follow up may not be long enough to capture such effect. Moreover, external validation for curative treatment criteria using separate data sets was not performed. Further large clinical trials of the three-day artesunate-mefloquine combination with different levels of initial parasite biomass is required. Also, only nausea and vomiting have been reported in patients and no other neurological deficits have been reported [18]. The accumulation of this drug exceeding 50 μM following standard treatment in some patients, however, would disrupt the function of neuronal calcium homeostasis and ER functions [30]. Results based on this simulation showed that there were not significantly different of predicted brain-concentration profiles between patients with and without adverse events following a standard treatment (p = 0.2734). Nevertheless, the RR of mefloquine concentrations over threshold of the disruption of neuronal calcium homeostasis for each regimen compared with standard treatment were reported for a risk-benefit assessment (All regimens are at risk of the disruption of neuronal calcium homeostasis).

## Conclusions

In conclusion, clinical use of a three-day artesunate-mefloquine (regimen-I) is suitable only when the IC_50_ of *P*. *falciparum* isolates is lower than 36 nM. With the resistant strains (IC_50_ up to 48 nM), two proposed regimens (III and IV) are preferable under DOT therapy or supervised medication. Otherwise, other ACT regimens should be replaced as the clinical efficacy would be dramatically decreased if patients’ adherence to medication is poor. For mefloquine-sensitive parasite, all regimens should provide satisfactory efficacy if the IC_50_ is lower than 23.19 nM. With decreased parasite sensitivity (IC_50_ close to 24 nM), a three-day artesunate-mefloquine is recommended since the efficacy of all proposed regimens would be extremely reduced to lower than 60%.

## Supporting information

S1 File Contains all the supporting tables and figures. (DOCX)
