# Supplementary material for: Prediction of improved antimalarial chemotherapy of artesunate-mefloquine in combination with mefloquine sensitive and resistant Plasmodium falciparum malaria
Source: PLoS One. 2023 Feb 23;18(2):e0282099. doi: 10.1371/journal.pone.0282099 (PMC9949628; doi:10.1371/journal.pone.0282099)

**Supplementary material**

**Table S1.**  OFV, AIC, BIC, and BICc criteria

| Parameters | Sensitive mefloquine | Resistant mefloquine | Artesunate | DHA |
| --- | --- | --- | --- | --- |
| OFV | 14425.52 | 3257.49 | -9836.61 | -1066.77 |
| AIC | 14457.52 | 3289.49 | -9798.61 | -968.77 |
| BICc | 14514.25 | 3297.25 | -9736.46 | -906.65 |
| BIC | 14491.03 | 3320.92 | -9750.70 | -920.86 |

| Mefloquine (sensitive) | | | | Mefloquine (resistance) | | |
| --- | --- | --- | --- | --- | --- | --- |
| Fixed effects | | | | | | |
| Parameters | Mean | SE | %RSE | Mean | SE | %RSE |
| T_k__pop | 6.05 | 0.47 | 7.78 | 9.83 | 1.85 | 18.8 |
| V_pop | 155915.5 | 6232.5 | 4.00 | 162500.0 | 20800 | 12.9 |
| k_pop | 0.0028 | 0.00025 | 9.02 | 0.0029 | 0.0005 | 17.8 |
| R_0__pop | 7649998.41 | 11.13 | 0.000146 | 10600000 | 2060000 | 19.4 |
| k_out__pop | 0.13 | 0.0044 | 3.48 | 0.0902 | 0.00661 | 7.33 |
| IC_50__pop | 3.41 | 2.12 | 62.1 | 29.5 | 11.6 | 39.1 |
| Error model parameters | | | | | | |
| a | 46.21 | 13.28 | 28.7 | 161 | 73.1 | 45.4 |
| b | 0.13 | 0.011 | 8.40 | 0.162 | 0.046 | 28.3 |
| aParasite | 102394.71 | 19051.99 | 18.6 | 192000 | 69100 | 35.9 |
| bParasite | 0.39 | 0.03 | 7.62 | 0.295 | 0.0629 | 21.3 |

**Table S2** PK/PD parameters

**Table S3**. PK parameters

| Artesunate | | | | Dihydroartemisinin (DHA) | | |
| --- | --- | --- | --- | --- | --- | --- |
| Fixed effects | | | | | | |
| Parameters | Mean | SE | %RSE | Mean | SE | %RSE |
| Ktr_pop | 14.9 | 1.44 | 9.69 | 1.18 | 0.19 | 16.3 |
| Mtt_pop | 1.36 | 0.085 | 6.21 | 3.17 | 0.069 | 2.19 |
| k_a__pop | 31.74 | 4.96 | 15.6 | 12.75 | 1.08 | 8.47 |
| Cl_pop | 50909.09 | 17472.02 | 34.3 | 10238.33 | 2520.41 | 24.60 |
| V_1__pop | 84.89 | 40.45 | 47.6 | 1.82 | 0.69 | 37.7 |
| Q_2__pop | 12.33 | 1.69 | 14.9 | 59.56 | 29.88 | 50.2 |
| V_2__pop | 0.096 | 0.015 | 15.9 | 0.083 | 0.013 | 15.9 |
| Q_3__pop | 9.54 | 1.06 | 11.1 | 23 | 4.57 | 19.90 |
| V_3__pop | 0.24 | 0.03 | 12.5 | 0.75 | 0.22 | 29.2 |
| Error model parameters | | | | | | |
| b | 0.89 | 0.061 | 6.84 | 0.75 | 0.044 | 5.79 |

**Table S4.** Treatment efficacy, relative risk, number-need to treat (NNT) of different proposed regimens with different IC_50_ values and adherences in patients with resistant mefloquine

| Regimen | %Efficacy | Relative risk | NNT | P-value |
| --- | --- | --- | --- | --- |
| 100% of adherence | | | | |
| 24 nM (1-fold) | | | | |
| 1 | 57.3 |  |  |  |
| 2 | 98.3 | 0.58 (0.55-0.61) | 2.44 (2.27-2.64) | <0.001 |
| 3 | 85.5 | 0.67 (0.63-0.71) | 3.55 (3.11-4.10) | <0.001 |
| 4 | 81.10 | 0.64 (0.60-0.67) | 3.06 (2.74-3.44) | <0.001 |
| 5 | 98.4 | 0.58 (0.55-0.61) | 2.43 (2.25-2.64) | <0.001 |
| 36 nM (1.5-fold) | | | | |
| 1 | 40.4 |  |  |  |
| 2 | 96.7 | 0.42 (0.39-0.45) | 1.76 (1.67-1.88) | <0.001 |
| 3 | 74.4 | 0.59 (0.54-0.64) | 3.56 (3.08-4.18) | <0.001 |
| 4 | 68.5 | 0.54 (0.50-0.59) | 2.94 (2.62-3.34) | <0.001 |
| 5 | 96.9 | 0.42 (0.39-0.45) | 1.77 (1.67-1.88) | <0.001 |
| 48 nM (2-fold) | | | | |
| 1 | 26 |  |  |  |
| 2 | 93.8 | 0.28 (0.25-0.31) | 1.48 (1.41-1.54) | <0.001 |
| 3 | 64.8 | 0.40 (0.36-0.45) | 2.58 (2.33-2.87) | <0.001 |
| 4 | 55.5 | 0.47 (0.42-0.53) | 3.39 (2.96-3.94) | <0.001 |
| 5 | 94.5 | 0.28 (0.25-0.31) | 1.46 (1.39-1.53) | <0.001 |
| 72 nM (3-fold) | | | | |
| 1 | 9.2 |  |  |  |
| 2 | 87.0 | 0.11 (0.09-0.13) | 1.29 (1.24-1.33) | <0.001 |
| 3 | 44.8 | 0.21 (0.17-0.25) | 2.81 (2.54-3.12) | <0.001 |
| 4 | 36.4 | 0.25 (0.20-0.31) | 3.68 (3.25-4.22) | <0.001 |
| 5 | 88.1 | 0.10 (0.08-0.13) | 1.27 (1.22-1.31) | <0.001 |
| 110 nM (5-fold) | | | | |
| 1 | 1 |  |  |  |
| 2 | 71.7 | 0.014 (0.007-0.025) | 1.41 (1.36-1.47) | <0.001 |
| 3 | 21.1 | 0.05 (0.026-0.09) | 4.98 (4.40-5.76) | <0.001 |
| 4 | 12.9 | 0.08 (0.04-0.14) | 8.40 (7.10-10.44) | <0.001 |
| 5 | 70.6 | 0.014 (0.007-0.026) | 1.44 (1.37-1.50) | <0.001 |
| 70% of adherence | | | | |
| 24 nM (1-fold) | | | | |
| 1 | 57.3 |  |  |  |
| 3 | 80.8 | 0.71 (0.67-0.75) | 4.26 (3.63-5.11) | <0.001 |
| 4 | 71.1 | 0.81 (0.75-0.86) | 7.25 (5.54-10.40) | <0.001 |
| 36 nM (1.5-fold) | | | | |
| 1 | 40.4 |  |  |  |
| 3 | 68.1 | 0.59 (0.54-0.65) | 3.61 (3.12-4.25) | <0.001 |
| 4 | 56.5 | 0.72 (0.65-0.78) | 6.21 (4.87-8.50) | <0.001 |
| 48 nM (2-fold) | | | | |
| 1 | 26.0 |  |  |  |
| 3 | 57.2 | 0.45 (0.40-0.51) | 3.21 (2.82-3.69) | <0.001 |
| 4 | 43.9 | 0.65 (0.57-0.73) | 7.00 (5.45-9.74) | <0.001 |
| 72 nM (3-fold) | | | | |
| 1 | 9.2 |  |  |  |
| 3 | 36.4 | 0.25 (0.20-0.31) | 3.68 (3.25-4.22) | <0.001 |
| 4 | 24.5 | 0.38 (0.30-0.47) | 6.54 (5.38-8.32) | <0.001 |
| 110 nM (5-fold) | | | | |
| 1 | 1.0 |  |  |  |
| 3 | 8.1 | 0.12 (0.06-0.23) | 14.08 (11.22-19.50) | <0.001 |
| 4 | 3.3 | 0.30 (0.15-0.60) | 43.48 (27.55-116.60) | <0.001 |
| 50% of adherence | | | | |
| 24 nM (1-fold) | | | | |
| 1 | 57.3 |  |  |  |
| 3 | 71.8 | 0.80 (0.75-0.85) | 6.90 (5.34-9.68) | <0.001 |
| 4 | 63.1 | 0.91 (0.85-0.98) | 17.24 (9.84-68.06) | <0.001 |
| 36 nM (1.5-fold) | | | | |
| 1 | 40.4 |  |  |  |
| 3 | 60.2 | 0.67 (0.61-0.73 | 5.05 (4.13-6.45) | <0.001 |
| 4 | 46.0 | 0.88 (0.79-0.97) | 17.86 (9.99-82.12) | <0.001 |
| 48 nM (2-fold) | | | | |
| 1 | 26.0 |  |  |  |
| 3 | 46.8 | 0.56 (0.49-0.63) | 4.81 (4.00-6.00) | <0.001 |
| 4 | 34.3 | 0.76 (0.66-0.87) | 12.05 (8.07-23.55) | <0.001 |
| 72 nM (3-fold) | | | | |
| 1 | 9.20 |  |  |  |
| 3 | 24.4 | 0.38 (0.30-0.47) | 6.58 (5.41-8.39) | <0.001 |
| 4 | 15.6 | 0.59 (0.46-0.75) | 15.63 (10.70-29.06) | <0.001 |
| 110 nM (5-fold) | | | | |
| 1 | 1.0 |  |  |  |
| 3 | 13.30 | 0.075 (0.04-0.14) | 8.13 (6.90-10.05) | <0.001 |
| 4 | 7.20 | 0.14 (0.07-0.26) | 16.13 (12.60-23.21) | <0.001 |
| 30% of adherence | | | | |
| 24 nM (1-fold) | | | | |
| 1 | 57.3 |  |  |  |
| 3 | 60.2 | 0.95 (0.88-1.02) | 34.48 (13.73-α) | NS (p=0.2) |
| 4 | 46.3 | 1.24 (1.14-1.35) | 9.09 (6.50-15.26) | <0.001 |
| 36 nM (1.5-fold) | | | | |
| 1 | 40.4 |  |  |  |
| 3 | 44.3 | 0.91 (0.82-1.01) | 25.64 (12.05-α) | NS (p=0.09) |
| 4 | 32.5 | 1.24 (1.10-1.39) | 12.99 (8.37-29.33) | <0.001 |
| 48 nM (2-fold) | | | | |
| 1 | 26.0 |  |  |  |
| 3 | 30.7 | 0.85 (0.74-0.97) | 21.28 (11.47-143.2) | NS (p=0.02) |
| 4 | 20.7 | 1.26 (1.07-1.47) | 18.87 (11.04-65.84) | 0.006 |
| 72 nM (3-fold) | | | | |
| 1 | 9.20 |  |  |  |
| 3 | 13.10 | 0.70 (0.55-0.90) | 25.64 (14.86-94.31) | 0.007 |
| 4 | 7.0 | 1.31 (0.97-1.77) | 45.45 (21.35-α) | NS (p=0.08) |
| 110 nM (5-fold) | | | | |
| 1 | 1 |  |  |  |
| 3 | 2.8 | 0.36 (0.18-0.72) | 55.56 (32.60-230.8) | 0.005 |
| 4 | 1.7 | 0.59 (0.28-1.26) | 142.9 (54.92-α) | NS (p=0.24) |

**Table S5.** Treatment efficacy, relative risk, number-need to treat (NNT) of different proposed regimens with different IC_50_ values and adherences in patients with sensitive mefloquine

| Regimen | %Efficacy | Relative risk | NNT | P-value |
| --- | --- | --- | --- | --- |
| 6 nM (0.25-fold) | | | | |
| 1 | 92.7 |  |  |  |
| 2 | 86.7 | 1.07 (1.04-1.10) | 15.63 (10.91-27.35) | <0.001 |
| 3 | 86.1 | 1.08 (1.05-1.11) | 15.15 (10.67-26.0) | <0.001 |
| 4 | 81.9 | 1.13 (1.09-1.17) | 9.26 (7.26-12.74) | <0.001 |
| 5 | 87.8 | 1.05 (1.03-1.09) | 20.41 (13.18-44.73) | <0.001 |
| 12 nM (0.5-fold) | | | | |
| 1 | 84.5 |  |  |  |
| 2 | 75.4 | 1.12 (1.07-1.17) | 10.99 (7.91-18.08) | <0.001 |
| 3 | 73.3 | 1.15 (1.10-1.21) | 8.93 (6.76-13.21) | <0.001 |
| 4 | 65.8 | 1.28 (1.22-1.35) | 5.35 (4.45-6.71) | <0.001 |
| 5 | 76.5 | 1.11 (1.06-1.15) | 12.5 (8.68-22.40) | <0.001 |
| 13.10 nM (0.54-fold) | | | | |
| 1 | 83.5 |  |  |  |
| 2 | 73.4 | 1.14 (1.09-1.19) | 9.90 (7.28-15.53) | <0.001 |
| 3 | 71.5 | 1.17 (1.11-1.22) | 8.33 (6.38-12.06) | <0.001 |
| 4 | 63.0 | 1.32 (1.26-1.40) | 4.88 (4.11-6.02) | <0.001 |
| 5 | 75.1 | 1.11 (1.06-1.16) | 11.9 (8.34-20.90) | <0.001 |
| 19.39 nM (0.8-fold) | | | | |
| 1 | 77.1 |  |  |  |
| 2 | 65.0 | 1.18 (1.12-1.26) | 8.26 (6.22-12.40) | <0.001 |
| 3 | 61.6 | 1.25 (1.18-1.33) | 6.45 (5.12-8.76) | <0.001 |
| 4 | 53.7 | 1.44 (1.34-1.54) | 4.27 (3.64-5.20) | <0.001 |
| 5 | 64.6 | 1.19 (1.13-1.26) | 8.0 (6.07-11.82) | <0.001 |
| 23.19 nM (0.96-fold) | | | | |
| 1 | 73.9 |  |  |  |
| 2 | 60.2 | 1.23 (1.15-1.31) | 7.30 (5.61-10.50) | <0.001 |
| 3 | 56.7 | 1.30 (1.22-1.39) | 5.81 (4.69-7.70) | <0.001 |
| 4 | 49.7 | 1.49 (1.38-1.60) | 4.13 (3.53-5.01) | <0.001 |
| 5 | 60.6 | 1.22 (1.15-1.30) | 7.52 (5.75-10.95) | <0.001 |

**Figure S1.** A comparison between predicted mefloquine whole-blood concentration and clinically observed data in patients with sensitive mefloquine


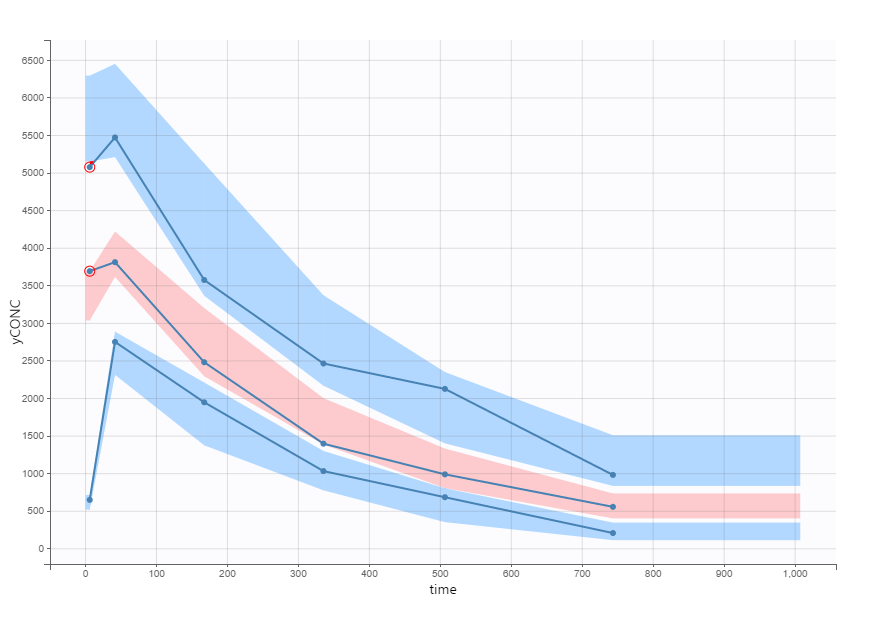


**Figure S2**. A comparison between predicted parasite density and clinically observed parasite density in patients with sensitive mefloquine.


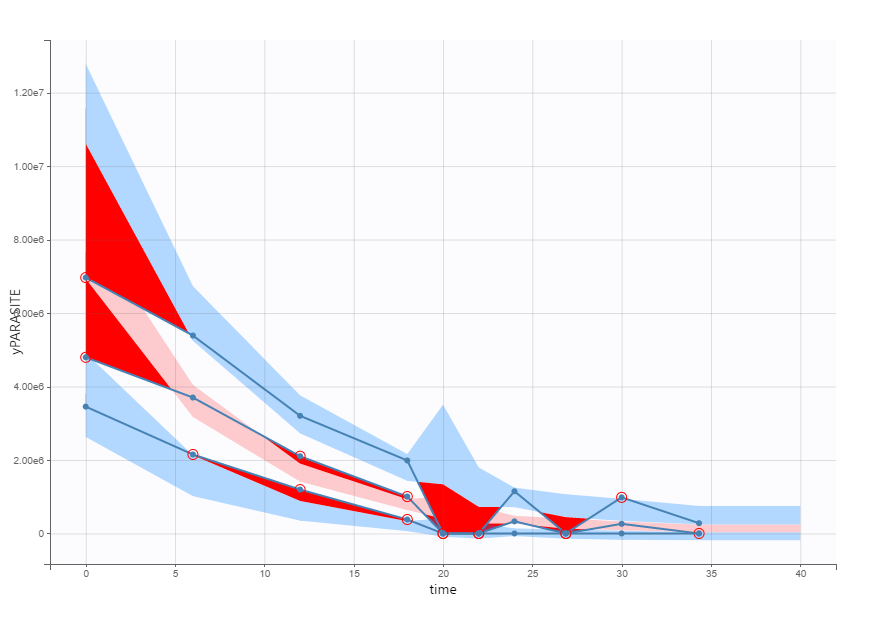


**Figure S3.** Observed versus predicted mefloquine whole-blood concentration in patients with sensitive mefloquine

**
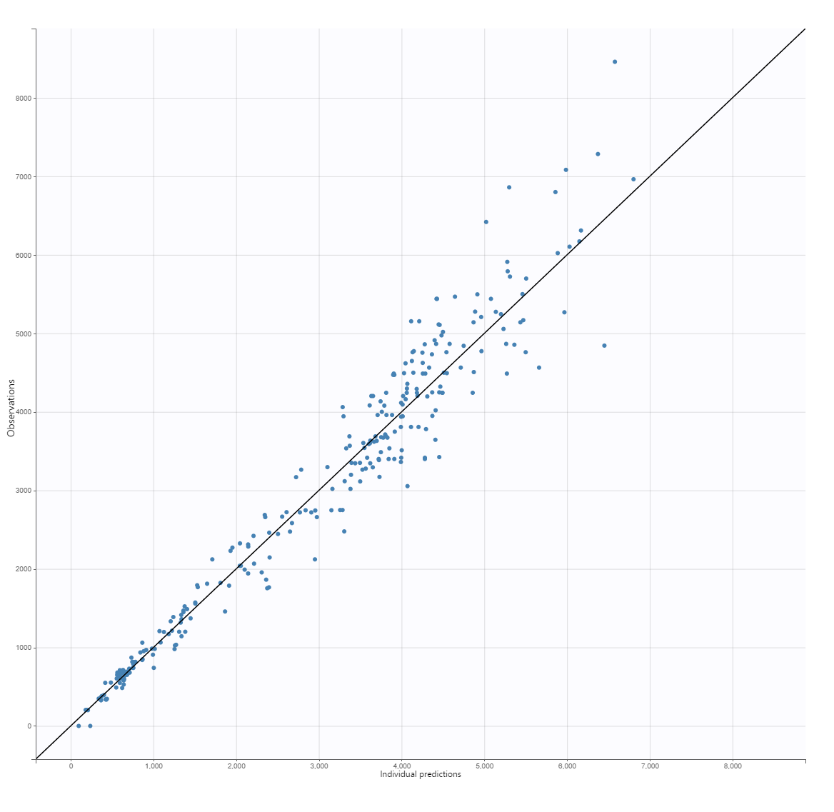
**

**Figure S4**. Residual scatter plot of mefloquine whole-blood concentration in patients with sensitive mefloquine

**
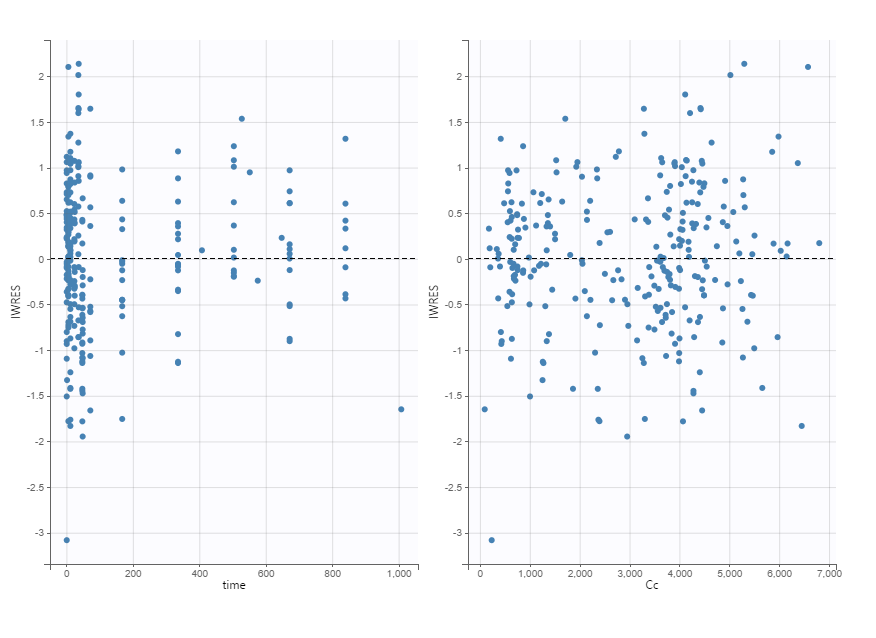
**

**Figure S5.** Observed versus predicted parasite density in patients with sensitive mefloquine

**
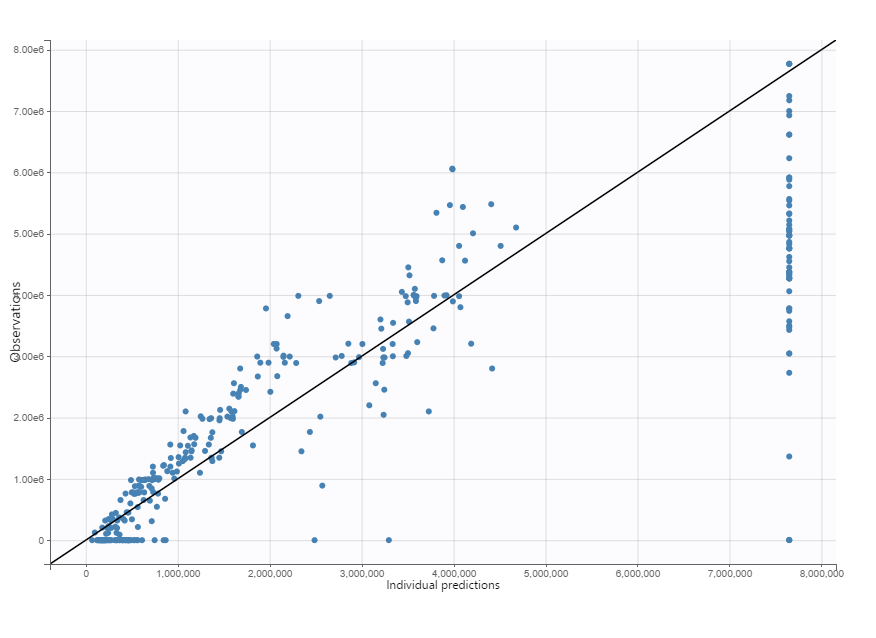
**

**Figure S6.** Residual scatter plot of parasite density in patients with sensitive mefloquine

**
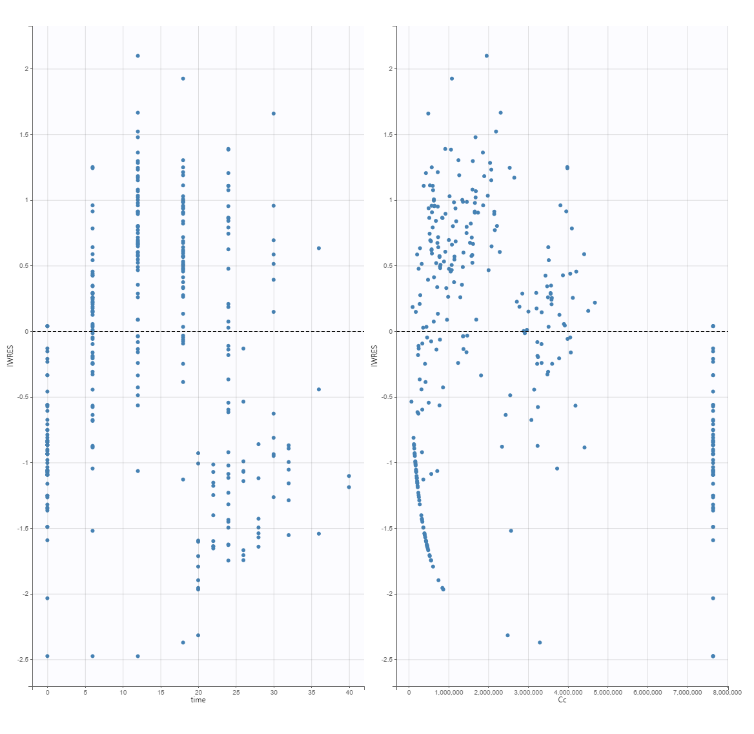
**

**Figure S7**. A comparison between predicted mefloquine whole-blood concentration and clinically observed data in patients with resistant mefloquine


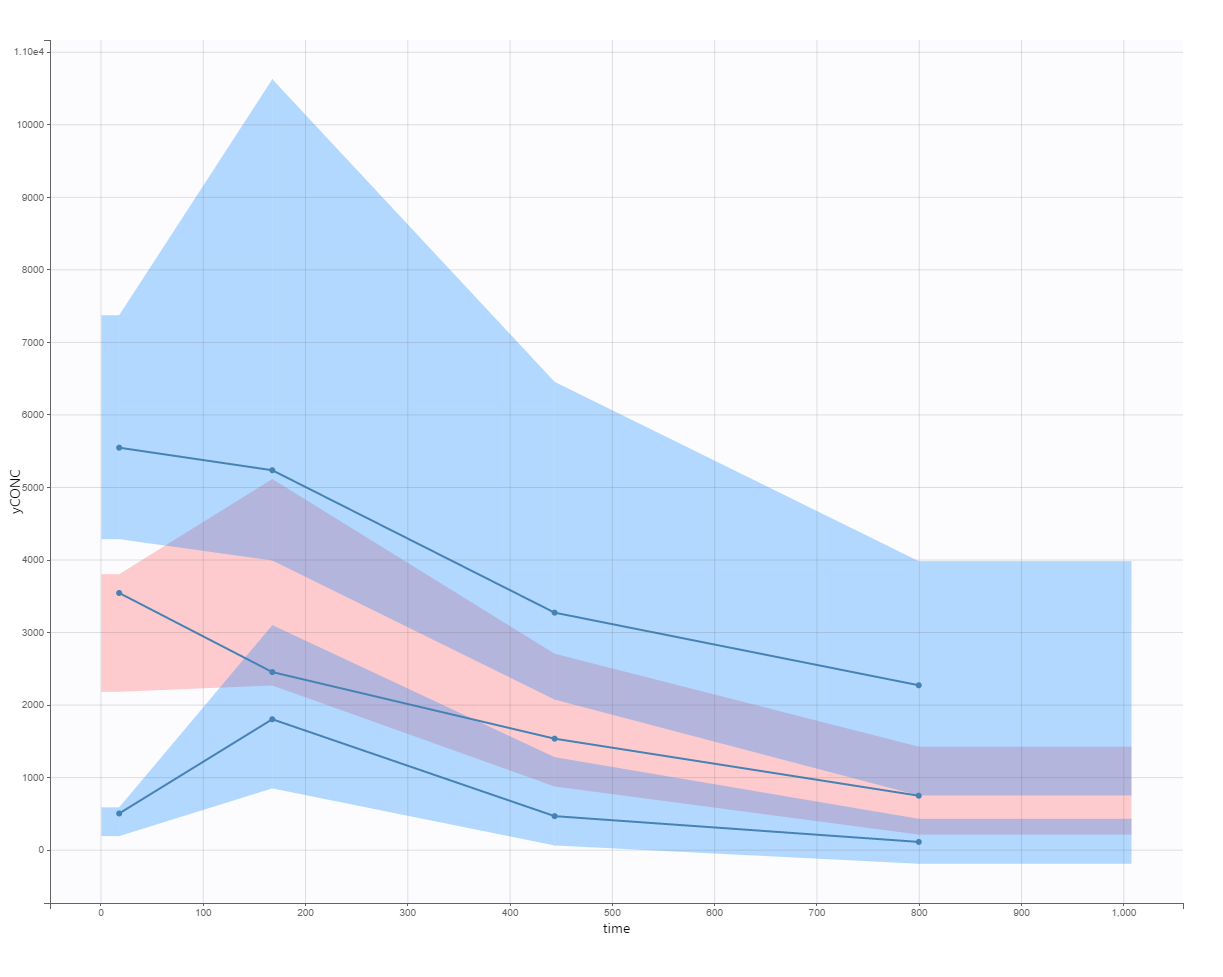


**Figure S8**. A comparison between predicted parasite density and clinically observed parasite density in patients with resistant mefloquine.


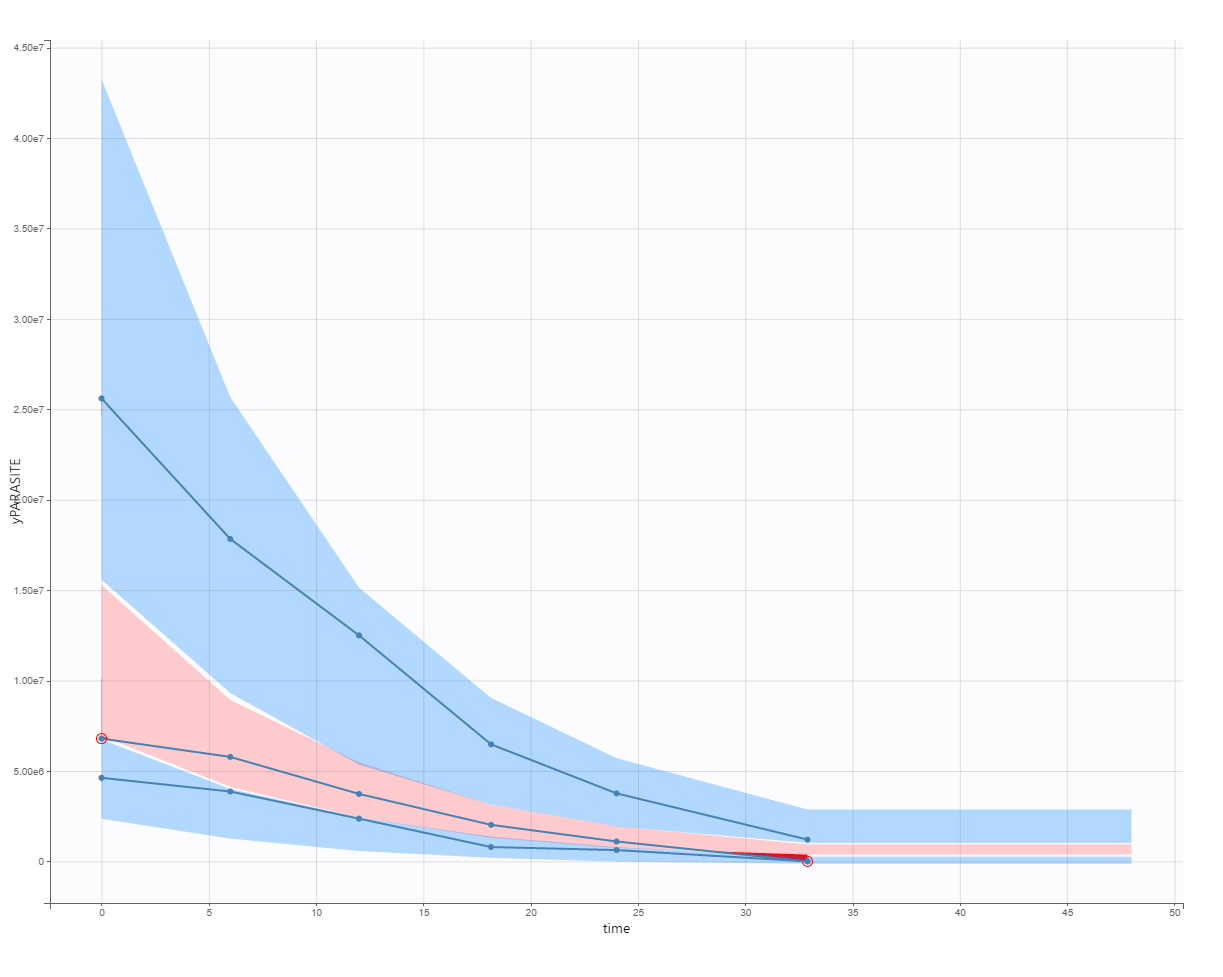


**Figure S9.** Observed versus predicted mefloquine whole-blood concentration in patients with resistant mefloquine.

**
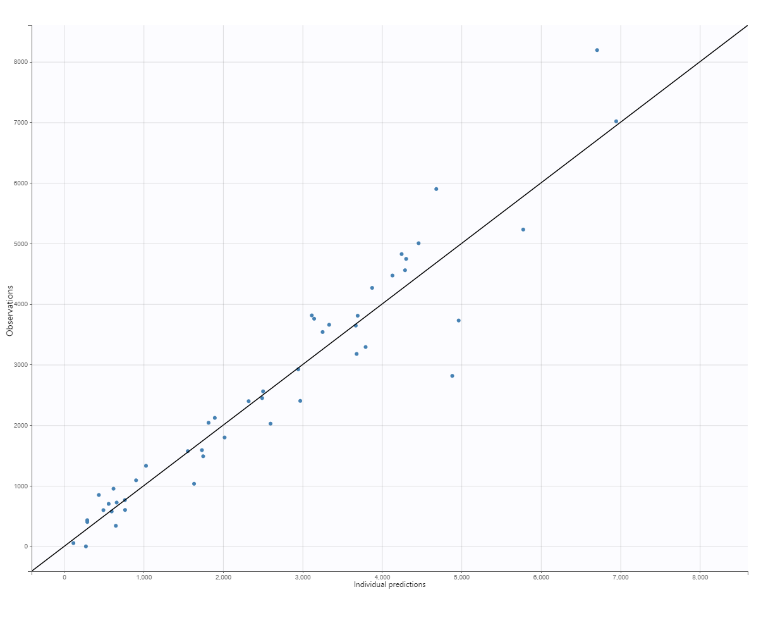
**

**Figure S10.** Residual scatter plot of mefloquine whole-blood concentration in patients with resistant mefloquine

**
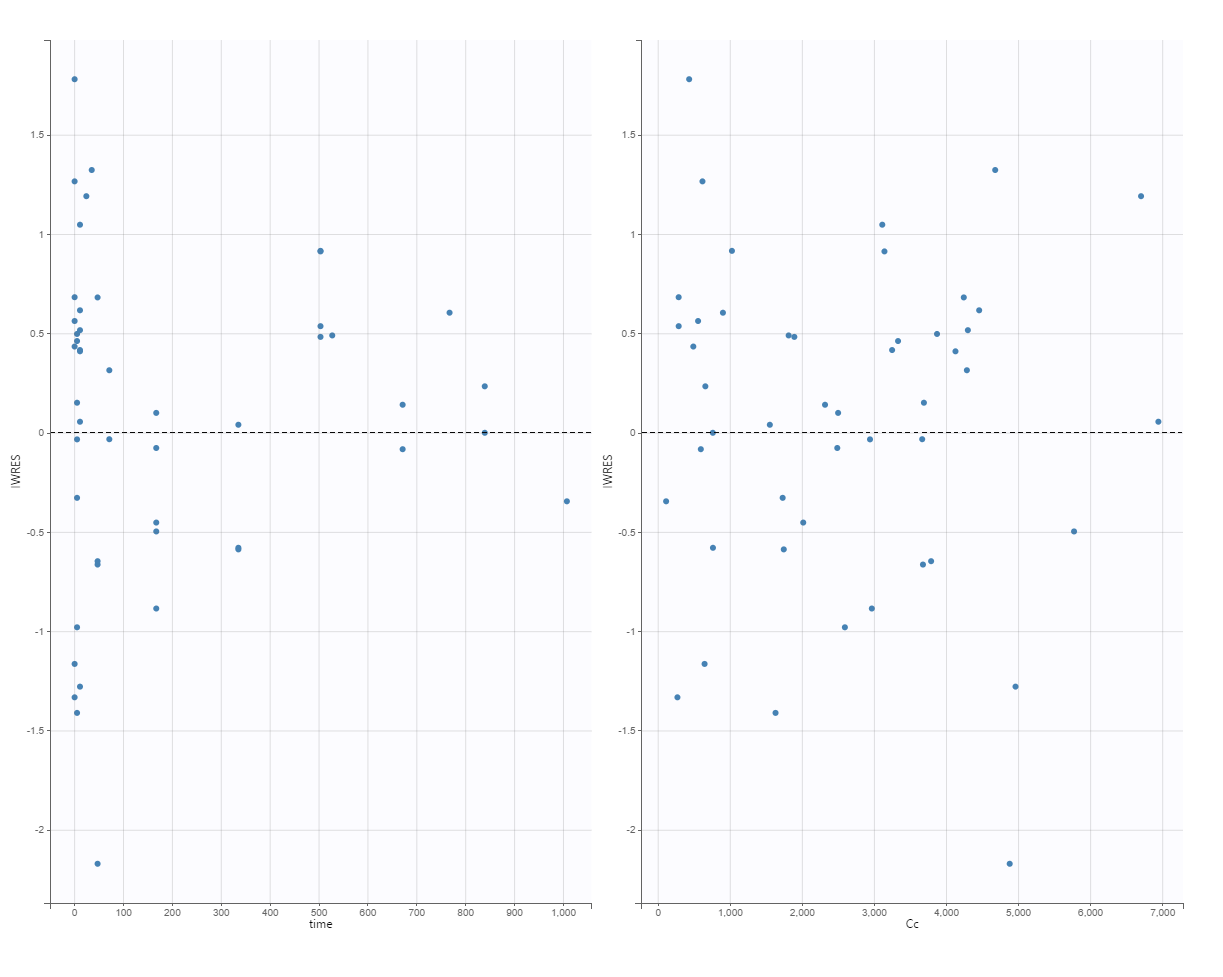
**

**Figure S11.** Observed versus predicted parasite density in patients with resistant mefloquine.

**
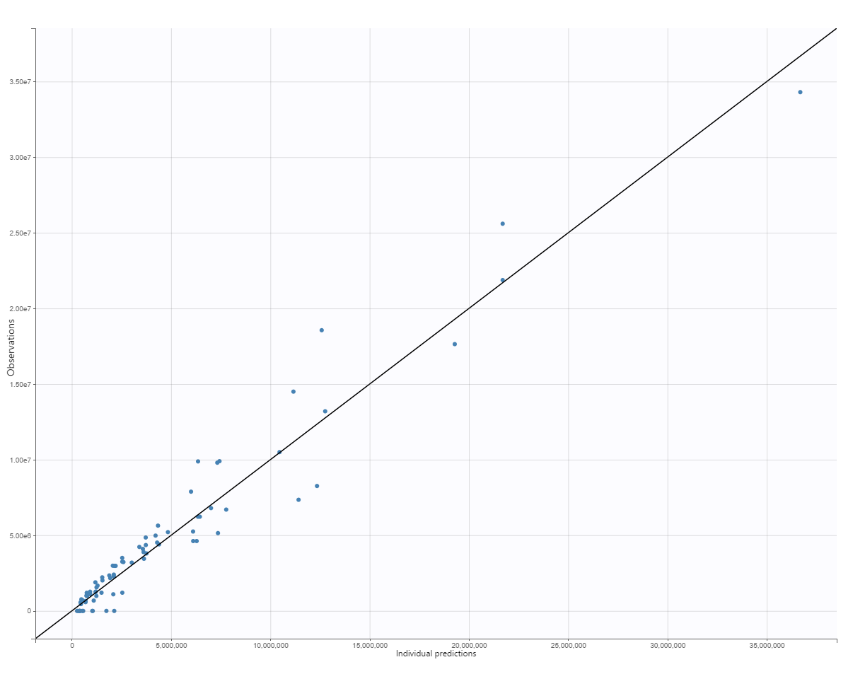
**

**Figure S12.** Residual scatter plot of parasite density in patients with resistant mefloquine.

**
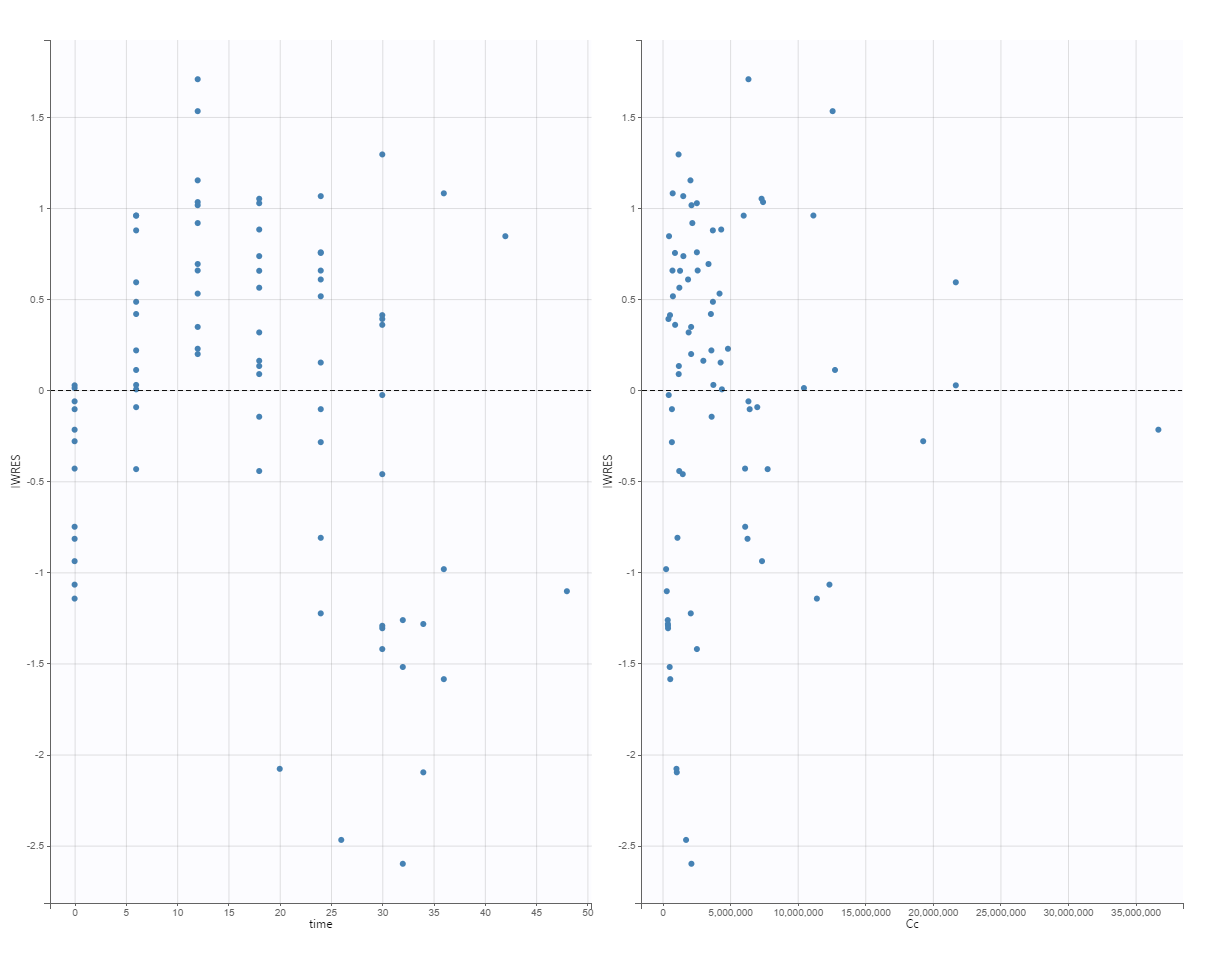
**

**Figure S13.** A comparison between predicted artesunate plasma-concentration and clinically observed data in patients.

**
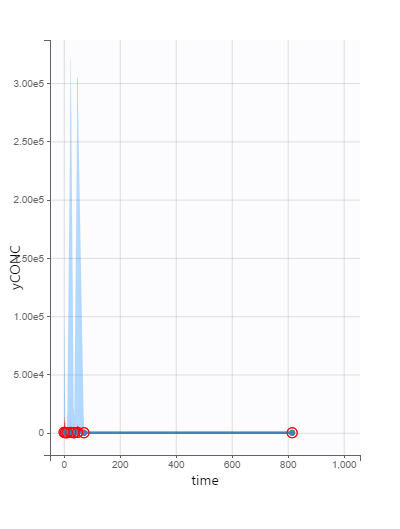
**

**Figure S14.** Observed versus predicted artesunate plasma-concentration in patients.

**
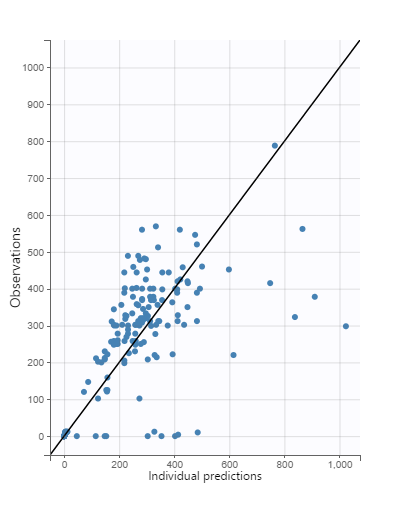
**

**Figure S15.** Residual scatter plot of artesunate plasma-concentration in patients.

**
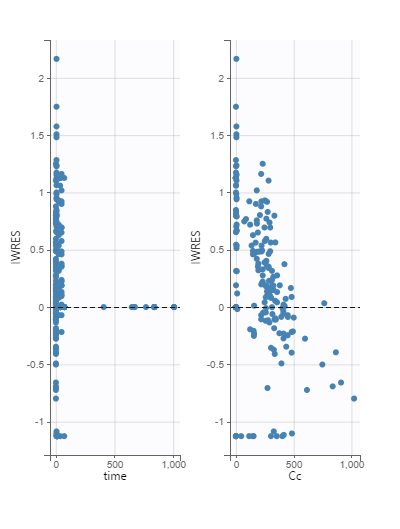
**

**Figure S16.** A comparison between predicted dihydroartemisinin plasma-concentration and clinically observed data in patients.

**
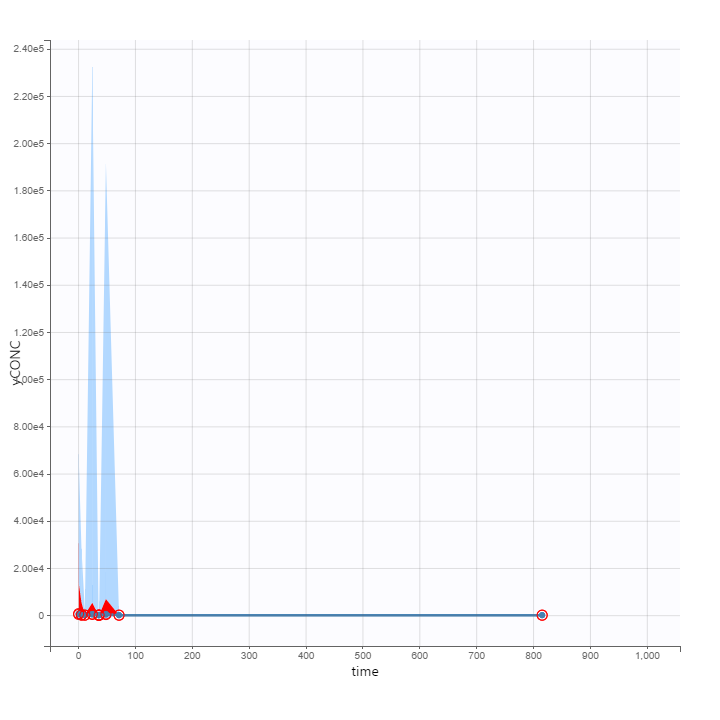
**

**Figure S17.** Observed versus predicted dihydroartemisinin plasma-concentration in patients**.**

**
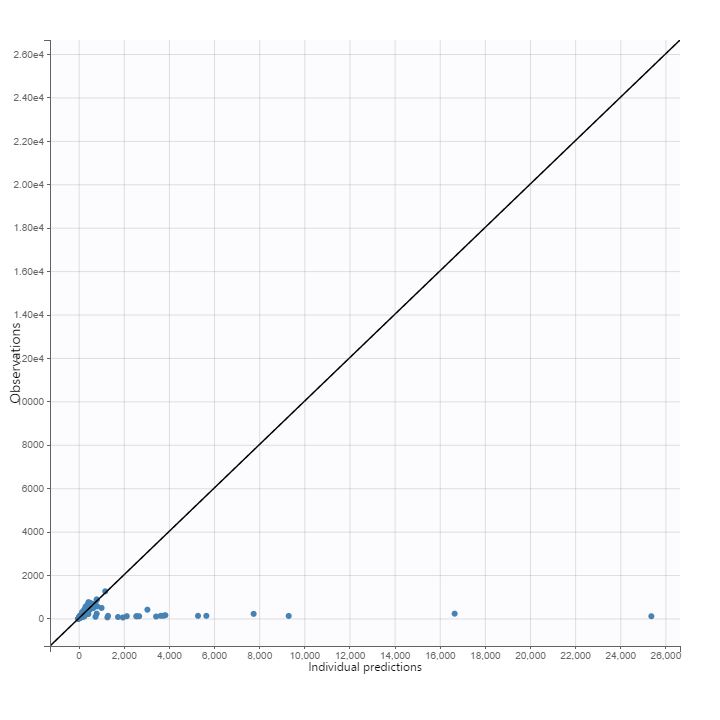
**

**Figure S18.** Residual scatter plot of dihydroartemisinin plasma-concentration in patients.

**
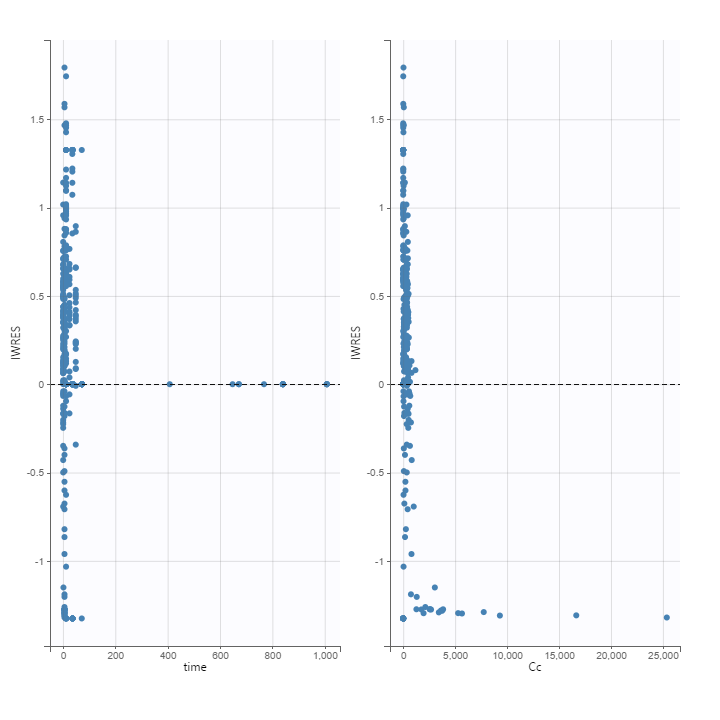
**

**Figure S19** Comparisons of relative risk of different proposed regimens and IC_50_ values with 100% of adherence in patients with resistant mefloquine**.**

**
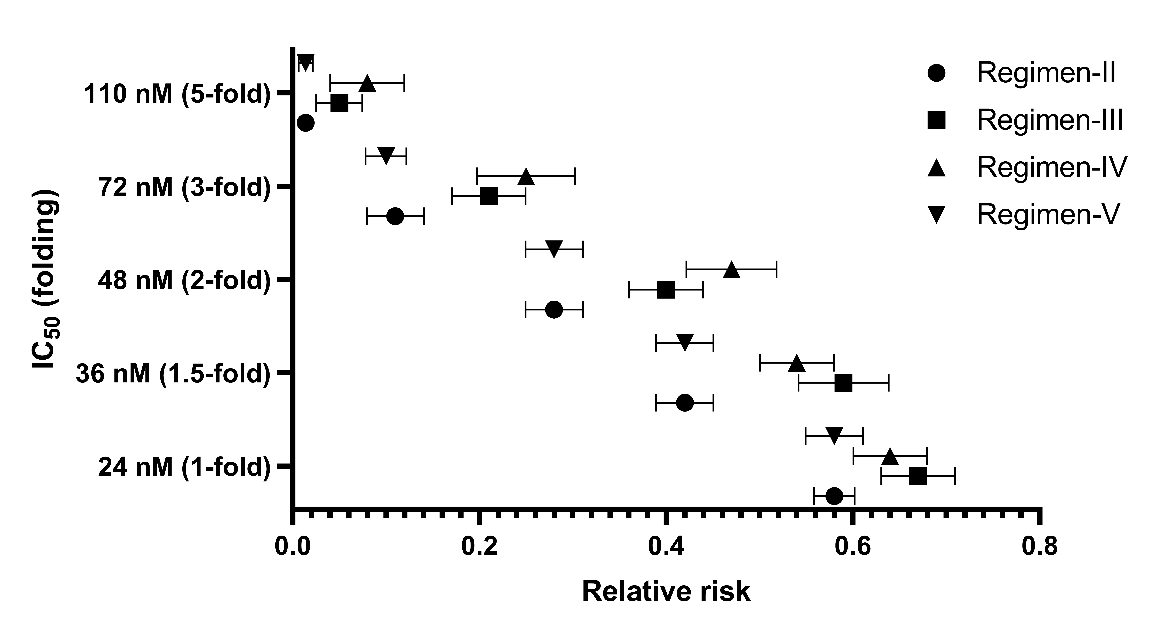
**

**Figure S20** Comparisons of number-need to treat of different proposed regimens and IC_50_ values with 100% of adherence in patients with resistant mefloquine**.**

**
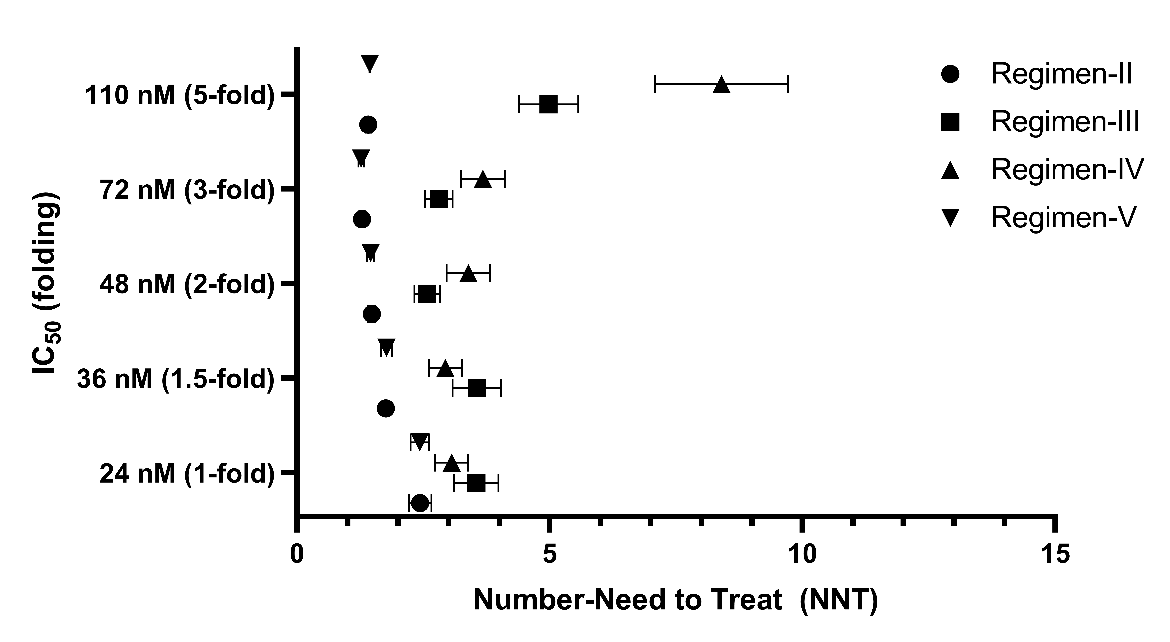
**

**Figure S21.** Comparisons of relative risk of two proposed regimens (III and IV) between 100% of adherence and 30% of adherence in patients with resistant mefloquine.


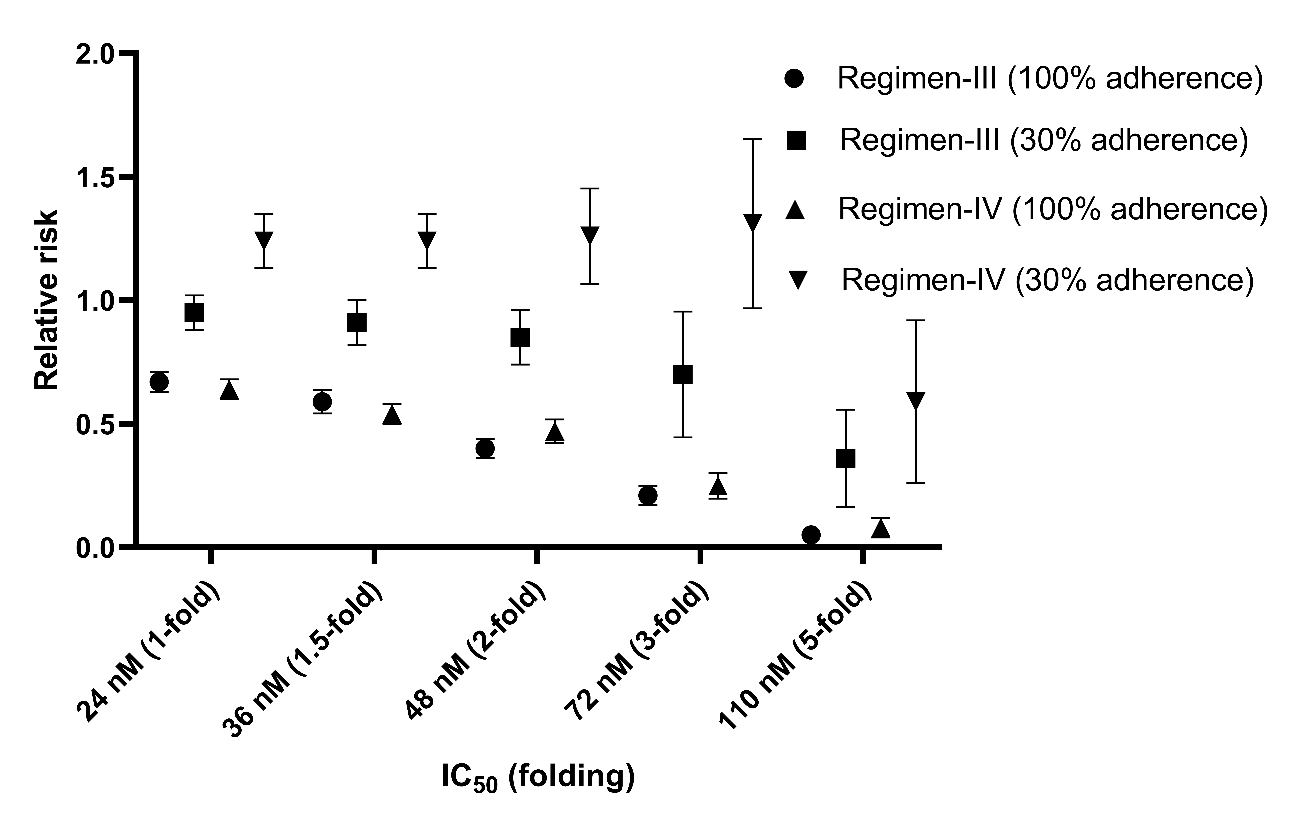

Supplement: S1 File — (DOCX) [file pone.0282099.s001.docx]
